# Authoritarian attitudes and the perceived scientific legitimacy of anthroposophic medicine: A survey of attitudes on complementary and alternative medicine in Austria

**DOI:** 10.1371/journal.pone.0348672

**Published:** 2026-06-17

**Authors:** Nikolas Reisecker, Julia Kuta, Levin Wieninger, Sebastian R. Leonard, Valentina Schmolik, Anita Rieder, Tanja Stamm, Edzard Ernst, Jutta Hübner, Harald H. Sitte, Valentin Ritschl

**Affiliations:** 1 Center for Physiology and Pharmacology, Institute of Pharmacology, Medical University of Vienna, Wien, Austria; 2 Institute for Outcomes Research, Center for Medical Data Science, Medical University of Vienna, Wien, Austria; 3 Ludwig Boltzmann Institute for Arthritis and Rehabilitation, Vienna, Austria; 4 Center for Public Health, Department for Social and Preventive Medicine, Medical University of Vienna, Vienna, Austria; 5 University of Exeter, United Kingdom; 6 Klinik für Innere Medizin II, Universitätsklinikum Jena, Germany; 7 Hourani Center for Applied Scientific Research, Al-Ahliyya Amman University, Amman, Jordan; 8 AddRess Centre for Addiction Research and Science, Medical University of Vienna, Vienna, Austria; Rehabilitative Hospital Affiliated to Fujian University of Traditional Chinese Medicine: Fujian University of Traditional Chinese Medicine Affiliated Rehabilitative Hospital, CHINA

## Abstract

**Objectives:**

This study investigates prevalence, usage, and perceived scientific legitimacy of anthroposophic medicine (AM) a prominent form of complementary and alternative medicine (CAM). It further explores associations with socioeconomic and psychological factors, with particular attention to authoritarian orientation, as measured by the KSA-3 scale and low ambiguity tolerance.

**Design/Setting:**

A cross-sectional online survey was conducted among the Austrian population assessing sociodemographic characteristics, healthcare utilization, attitudes toward conventional medicine, AM, and CAM, as well as selected psychological variables.

**Participants:**

A total of 429 individuals completed the survey. To enhance representativeness, the sample was post-stratified according to recent election results.

**Outcome Measures:**

Regression models and appropriate statistical tests, selected based on data distribution and scale level, were used to evaluate associations between sociodemographic factors, psychological characteristics, and the use of and attitudes toward AM and CAM.

**Results:**

Individuals over 50 showed higher CAM preference (p = 0.044), and were more likely to have used AM at least once (p = 0.083). Similarly, women showed higher CAM preferences (p = 0.004) and a higher likelihood of AM use (p < 0.001). Participants who viewed AM as scientific tended to be older (p < 0.001), have a higher income (p < 0.001), and resided more often in rural areas (p = 0.012). Psychologically, lower ambiguity tolerance and stronger perceived health control correlated with a stronger belief in AM’s scientific legitimacy (p < 0.05). Higher authoritarian orientation was also significantly associated with an increased belief in AM as scientific and a preference for eminence-based approaches (p < 0.001).

**Conclusions:**

In our sample, we demonstrate how differences in specific socioeconomic and psychological factors correspond to differences in attitudes toward CAM and AM. These findings represent a promising foundation for more interdisciplinary research aimed at uncovering the underlying drivers of AM and CAM utilization.

## Introduction

Anthroposophic medicine (AM) is often described as an integrative medical system, tracing its origins to the early 20th century [1,2]. Founded by Austrian esotericist Rudolf Steiner and Dutch physician Ita Wegman, they sought to develop a therapeutic approach that extends beyond the physical dimensions of human health to include both the spiritual and psychological [3]. In practice, anthroposophic medicine combines various treatments, including phytotherapeutic remedies (e.g., mistletoe therapy) [4], eurythmy (a movement-based therapy) [5], rhythmic massage [6], and anthroposophic art therapy [7], all of which are believed to promote holistic well-being. Beyond its therapeutic claims, anthroposophy encompasses a broader cultural framework which includes Waldorf education, biodynamic agriculture, and a range of lifestyle products, contributing to its sustained visibility. AM has been integrated into national healthcare systems in several European countries [8] and is offered as a recognized qualification in medical training programs in Austria, Germany, and Switzerland [9–11]. Its appeal has been attributed to the emphasis on treating patients as whole persons, addressing spiritual and psychological dimensions of health alongside the physical, a framing that resonates with broader trends in patient-centered care. Despite limited evidence for its therapeutic effectiveness [12–14], the movement claims 52,000 members from national societies across 50 countries [15].

The epistemological foundations of AM diverge fundamentally from those of modern science. Steiner’s “spiritual science” (“*Geisteswissenschaft*”) posits a form of supersensible cognition, cultivated through meditative practice, that purportedly grants access to dimensions of reality beyond empirical observation [16,17]. Within this framework, Steiner’s own writings function not as hypotheses subject to falsification but as foundational truths derived from higher perception, creating a self-referential epistemic structure resistant to external critique [18,16]. Proponents often position AM as legitimate science, yet its core claims (concerning etheric bodies, formative forces, and karmic processes) are not amenable to empirical testing as conventionally understood [17] and often contradict well-established scientific consensus, including those related to climate change [19]. While some within the movement acknowledge this fundamental incommensurability with scientific methodology [20], efforts to frame AM as scientifically valid persist; particularly in clinical and regulatory contexts. Rigorous systematic reviews have found no substantial evidence supporting the therapeutic effectiveness of anthroposophical treatments [12–14]. In response, ‌‌proponents have questioned the applicability of conventional evidence standards, arguing that healing operates on planes inaccessible to empirical verification and that evidence-based medicine represents an insufficient framework of inquiry [21,22].

The historical record reveals structural affinities between anthroposophy and authoritarian movements that illuminate this question. During the National Socialist period, AM and biodynamic agriculture found active support among high-ranking officials, most notably Rudolf Hess [23]. This compatibility was not incidental: anthroposophy’s hierarchical cosmology, its reliance on mystified epistemic authority, and its resistance to critical-rational inquiry share structural features with authoritarian modes of thought [23,24]. Steiner’s own writings contain explicit instances of racist and antisemitic ideation, reinforcing hierarchical conceptions of human development consonant with early twentieth-century *völkisch* ideology [25,26]. Writing in the aftermath of World War II, Adorno, Benjamin, and Bloch identified these structural parallels between ‌‌occult belief systems and authoritarian psychology [24,27,28]. Adorno argued that esoteric movements cultivate susceptibility to dogma and discourage critical self-reflection: what he termed “a symptom of the regression of consciousness” thereby providing fertile ground for authoritarian patterns of thinking [24]. AM’s persistence in contemporary healthcare thus cannot be understood solely as a matter of consumer preference or ideological anomaly. It reflects deeper patterns: the enduring appeal of esoteric belief systems, the institutional entrenchment of practices lacking empirical support, and unresolved tensions over epistemic authority in medicine. To move beyond merely evaluating AM’s scientific claims, the present study examines the social, psychological, and material conditions that sustain its appeal with particular attention to the role of authoritarian attitudes in shaping perceptions of AM’s scientific legitimacy. Methods

### Data collection and procedures

We conducted an online cross-sectional study using the ‘SoSciSurvey’ questionnaire platform to evaluate the general population. The questionnaire is available in German, with an English translation provided in the supplementary table 1.

Participants were recruited through online convenience sampling. The survey was distributed through multiple channels, including Facebook, WhatsApp, and Signal groups; individual contacts; the freely accessible newspaper *Heute* [German for “Today”]; and the *Newsflix* [https://www.newsflix.at/] news platform. The survey remained open from January 11, 2025, to February 16, 2025 (37 days).

For clarity, participants were provided the definitions of conventional, complementary, and alternative medicine at the start of the survey. If participants indicated that they were unfamiliar with anthroposophic medicine, they were automatically provided the relevant definition (see supplementary table 2). Participation was voluntary and anonymous. Before starting the questionnaire, participants were informed about the purpose of the study, the anonymous handling of the data, the voluntary nature of participation, and their right to discontinue participation at any time without disadvantage. Proceeding to the questionnaire after reading the study information was considered informed consent. No directly identifying personal data were collected.

### Survey instrument

The survey was organized into three main sections: (i) sociodemographic and economic data, such as age, gender, education level, income per month, and political orientation; (ii) health attitudes, such as attitudes toward evidence-based medicine and CAM, with a focus on anthroposophic medicine, and; (iii) psychological measures such as the World Health Organization Quality of Life Assessment (WHOQOL) [29], adapted health locus of control items based on the Multidimensional Health Locus of Control (MHLC) [30], items on Stress Management, Self-Efficacy [31], Prioritization of Health Optimization, Ambiguity Tolerance [32], and the Short Authoritarianism Scale (KSA-3) [33].

A broad frame regarding CAM was intentionally formulated to evoke an intuitive understanding among respondents, encompassing a wide range of practices without imposing strict definitional boundaries. This is addressed accordingly in our results and discussion. Concurrently, we incorporated explicit inquiries about anthroposophic medicine, assessing its use and associated beliefs about its scientific status, to enable a focused analysis of this specific modality. The survey also assessed participants’ use of conventional healthcare services, including preventive examinations and vaccination attitudes. Internal consistency was assessed using Cronbach’s α.

### Research questions

This survey explores the relationships between participants’ socioeconomic backgrounds, psychological characteristics, and attitudes toward CAM, anthroposophic medicine, and conventional medicine. Rather than testing specific hypotheses in an experimental sense, we investigate patterns and associations using descriptive statistics, regression models, and sensitivity analyses.

Based on previous research and theoretical considerations, we formulated the following research questions:

**Table:** 

Category	Description
**Healthcare Utilization and Medical Attitudes**	How do beliefs in (CAM) influence the use of conventional healthcare services, including preventive examinations and vaccination attitudes?
	Are individuals with chronic conditions more likely to seek CAM treatments?
	Does dissatisfaction with conventional healthcare lead to a greater preference for CAM therapies?
**Sociodemographic Factors Influencing CAM Attitudes and AM Attitudes**	What roles do sociodemographic factors (age, education, income, gender, political orientation) play in shaping attitudes toward CAM and AM?
	Is the use of anthroposophic medicine associated with a lower emphasis on scientific evidence and a stronger reliance on expert opinion?
	How does knowledge of evidence-based medicine vary between individuals with strong and weak CAM beliefs?
**Psychological Characteristics Influencing CAM Attitudes and AM Attitudes**	To what extent do psychological traits—such as quality of life, health locus of control, stress management, self-efficacy, health prioritization, ambiguity tolerance, and authoritarian attitudes—predict preferences for CAM over conventional medicine?
	To what extent do these psychological traits reveal any tendencies of using AM or believing it to be scientific?
**Authoritarian Attitudes and CAM Use**	Is there an association between authoritarian attitudes (low ambiguity tolerance and authoritarian leaning) and the likelihood of preferring CAM, spending more on CAM, using AM, or perceiving AM as scientific?
	Do individuals with stronger authoritarian tendencies favor eminence-based over evidence-based approaches?
	Are specific political party preferences linked to authoritarian attitudes and CAM beliefs?

### Statistical analysis

Descriptive statistics were used to summarize the data. Categorical variables were reported as absolute frequencies and percentages. Ordinal and metric variables were summarized using the mean and standard deviation (SD) and the median and interquartile range (IQR). These statistics were calculated for the overall study population and relevant subgroups.

To examine group differences, we applied appropriate statistical tests based on data distribution and scale level of the respective variables. Continuous variables were analyzed using independent-samples t-tests when comparing two groups and one-way analysis of variance when comparing more than two groups, provided that distributional assumptions were considered acceptable. For non-normally distributed or ordinal variables, Mann-Whitney U tests or Kruskal-Wallis tests were used as appropriate. Categorical variables were analyzed using chi-square tests of independence or Fisher’s exact tests when expected cell counts were small. Sociodemographic variables were treated according to their measurement level. Age was considered a continuous variable in regression models, whereas variables such as gender, education, residential setting, and income group were treated as categorical predictors.

: Additionally, we investigated which psychological characteristics (perceived quality of life, health locus of control, stress management, self-efficacy, prioritization of health optimization, ambiguity tolerance, and authoritarian attitudes) predict a preference for CAM, as intuitively understood by participants over conventional approaches. To assess these relationships, we conducted a multiple linear regression analysis., For regression models, we report regression coefficients, standard errors (SE), 95% confidence intervals (CI), p-values, and model fit indices (R²).

To account for potential sampling distortions, post-stratification weighting was applied based on respondents’ reported voting behavior. Inspection of the sample distribution revealed substantial deviations from the official distribution of party preferences in the Austrian National Council election, indicating that the sample was not representative of the Austrian voting population. Population targets were derived from the official results of the most recent Austrian National Council election. For each political category, a post-stratification weight was calculated as the ratio between the population proportion and the corresponding sample proportion (weight = population proportion/sample proportion). These weights were then assigned to individual observations and incorporated into regression analyses.

This procedure partially corrects for the over- or underrepresentation of specific political groups in the sample and improves the generalizability of the estimated associations. However, post-stratification weighting can only adjust for known imbalances and does not fully eliminate potential sampling bias. All tests were two-sided, and p-values < .05 were considered statistically significant.All statistical analyses and visualizations were conducted using R version 4.4.1 “Race for Your Life” (www.r-project.org)

### Ethics statements

#### Patient consent for publication.

Not applicable. This study used an anonymous survey without collecting identifiable data; therefore, individual consent for publication was not required.

### Ethics approval

Following correspondence with the chair of the ethics committee at the Medical University of Vienna, it was determined that formal ethics approval was not required due to the study design, and the study was exempted from further approvals. Informed consent was obtained from all participants prior to the survey via a checkbox selection.

## Results

The survey link was accessed 1,836 times, resulting in 868 interviews. Of these, 638 participants completed the questionnaire until the end. However, only 429 provided sufficient data—defined as having less than 20% missing responses and answering beyond the mandatory questions. Consequently, the final analysis was based on 429 participants.

Internal consistency of the multi-item measures was evaluated using Cronbach’s α. Reliability varied across the scales used in this study. Cronbach’s α was 0.52 for the WHOQOL item composite, 0.64 for Self-Efficacy, and 0.59 for Stress Management. Internal consistency was lower for MSTAT-II (α = 0.43) and the Short Authoritarianism Scale KSA-3 (α = 0.49). The adapted health locus of control item composites showed low internal consistency (α = 0.30 for the broader composite and α = 0.32 for the internal-control composite) and were therefore interpreted with caution in subsequent analyses.

### Demographic Analysis

The study population comprised 286 women and 143 men aged from 15 to over 65 years. Nearly half (49%) were in their twenties, with 29.6% aged 20–24 and 20.3% aged 25–29. Only a small proportion were teenagers (1.2% aged 15–19) or in the oldest age groups (approximately 10% aged 60 or older). The remaining participants (approximately 30%) were distributed across the intermediate age ranges of 30–59 years.

Regarding educational attainment, 25.2% (n = 108) had completed AHS with Matura as their highest form of education, 20.3% (n = 87) held a bachelor’s degree, and 19.1% (n = 82) had obtained a magister/master’s/diploma qualification. The remaining participants reported a range of other educational backgrounds, including vocational and lower-level qualifications.

**Fig 1:**
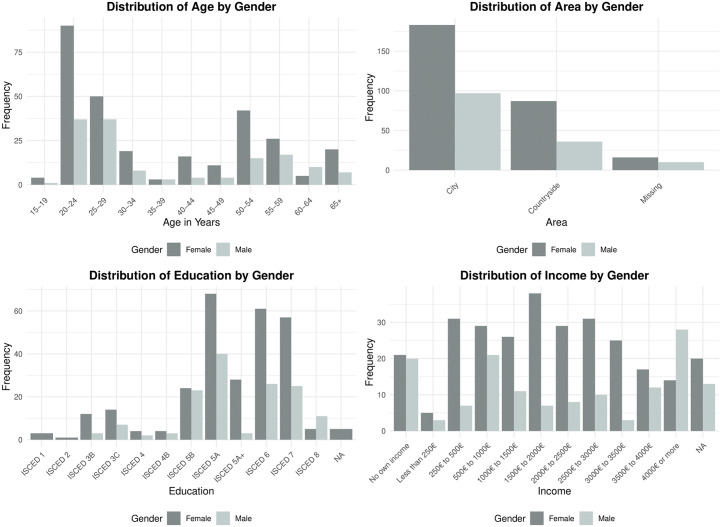
Demographic Block Diagrams of Respondents.

In terms of citizenship, the vast majority were Austrian (94.9%, n = 407), while 3.0% (n = 13) were German, 0.5% (n = 2) were Swiss, and 1.6% (n = 7) were from other countries. Detailed demographic characteristics of the study population, including statistical comparisons between male and female participants, are presented in Table 1 (Fig 1).

**Table 1:** Demographic data.

Variables	Level	Overall	Male	Female	p
**Numbers of Participants, n**		429	143	286	
***Age, n (%)***	15 to 19 years	5 (1.2)	1 (0.7)	4 (1.4)	0.063
	20 to 24 years	127 (29.6)	37 (25.9)	90 (31.5)	
	25 to 29 years	87 (20.3)	37 (25.9)	50 (17.5)	
	30 to 34 years	27 (6.3)	8 (5.6)	19 (6.6)	
	35 to 39 years	6 (1.4)	3 (2.1)	3 (1.0)	
	40 to 44 years	20 (4.7)	4 (2.8)	16 (5.6)	
	45 to 49 years	15 (3.5)	4 (2.8)	11 (3.8)	
	50 to 54 years	57 (13.3)	15 (10.5)	42 (14.7)	
	55 to 59 years	43 (10.0)	17 (11.9)	26 (9.1)	
	60 to 64 years	15 (3.5)	10 (7.0)	5 (1.7)	
	65 or older	27 (6.3)	7 (4.9)	20 (7.0)	
***Age grouped, n (%)***	15–25	132 (30.8)	38 (26.6)	94 (32.9)	0.394
	25–49	155 (36.1)	56 (39.2)	99 (34.6)	
	Older than 50	142 (33.1)	49 (34.3)	93 (32.5)	
**Location*, n (%)***	Countryside	123 (30.5)	36 (27.1)	87 (32.2)	0.346
	City	280 (69.5)	97 (72.9)	183 (67.8)	
**Education*, n (%)***	AHS with Matura (general secondary school with diploma)	108 (25.2)	40 (28.0)	68 (23.8)	0.002
	Other school	5 (1.2)	0 (0.0)	5 (1.7)	
	Bachelor’s	87 (20.3)	26 (18.2)	61 (21.3)	
	Professional Maturity Exam/Vocational Matura (evening school)	7 (1.6)	3 (2.1)	4 (1.4)	
	BHS with Matura (e.g., HTL, HAK, HBLA, etc.)	47 (11.0)	23 (16.1)	24 (8.4)	
	BMS (vocational school, e.g., HASCH)	15 (3.5)	3 (2.1)	12 (4.2)	
	Doctorate/ PhD	16 (3.7)	11 (7.7)	5 (1.7)	
	University-related educational institution or college	31 (7.2)	3 (2.1)	28 (9.8)	
	Apprenticeship, vocational school	21 (4.9)	7 (4.9)	14 (4.9)	
	Magister/ Master’s/ Diplom-Ingenieur/ University of Applied Sciences/ Medical Studies	82 (19.1)	25 (17.5)	57 (19.9)	
	Polytechnic	1 (0.2)	0 (0.0)	1 (0.3)	
	University entrance examination	6 (1.4)	2 (1.4)	4 (1.4)	
	Primary school or less, lower secondary school, or lower AHS	3 (0.7)	0 (0.0)	3 (1.0)	
**Education grouped*, n (%)***	Below Matura	133 (31.0)	37 (25.9)	96 (33.6)	0.267
	Matura (school leaving exam)	193 (45.0)	69 (48.3)	124 (43.4)	
	Above Matura	103 (24.0)	37 (25.9)	66 (23.1)	
***Income per month, n (%)***	I do not have my own income	41 (10.4)	20 (15.4)	21 (7.9)	<0.001
	Less than 250€	8 (2.0)	3 (2.3)	5 (1.9)	
	250-500€	38 (9.6)	7 (5.4)	31 (11.7)	
	500-1000€	50 (12.6)	21 (16.2)	29 (10.9)	
	1000-1500€	37 (9.3)	11 (8.5)	26 (9.8)	
	1500-2000€	45 (11.4)	7 (5.4)	38 (14.3)	
	2000-2500€	37 (9.3)	8 (6.2)	29 (10.9)	
	2500-3000€	41 (10.4)	10 (7.7)	31 (11.7)	
	3000-3500€	28 (7.1)	3 (2.3)	25 (9.4)	
	3500-4000€	29 (7.3)	12 (9.2)	17 (6.4)	
	4000€ or more	42 (10.6)	28 (21.5)	14 (5.3)	
**Income grouped*, n (%)***	Less than 1500 €	174 (43.9)	62 (47.7)	112 (42.1)	0.001
	1500–2999 €	123 (31.1)	25 (19.2)	98 (36.8)	
	More than 3000 €	99 (25.0)	43 (33.1)	56 (21.1)	

P-values were derived from chi-square tests of independence or Fisher’s exact tests, as appropriate. Fisher’s exact tests were used for detailed age, education, and income categories (age: p = 0.063; education: p = 0.002; income: p < 0.001). Chi-square tests were used for grouped age (χ²(2) = 1.86, p = 0.394), location (χ²(1) = 0.89, p = 0.346), education grouped (χ²(2) = 2.64, p = 0.267), and income grouped (χ²(2) = 14.39, p < 0.001).

**Fig 2:**
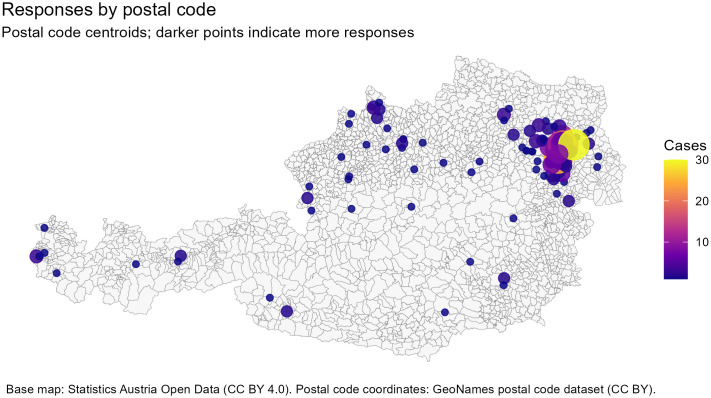
Geographic Distribution of Survey Responses in Austria. A map of Austria highlights communities (Gemeinden) in proportion to the number of survey responses received, illustrating regional variations in participation. DE = Germany; CZ = Czech Republic; SK = Slovakia; HU = Hungary; SI = Slovenia; IT = Italy; CH = Switzerland.

Responses were obtained from a broad range of regions across Austria; however, the sample exhibited a notable urban bias, with a disproportionate number of respondents from Vienna (Fig 2).

Approximately one in ten participants (10.4%) reported having no personal income. Among those who reported earnings, income levels covered a wide range. The most frequently reported brackets were between €500-€1,000 (12.6%), €1,500-€2,000 (11.4%), and €2,500-€3,000 (10.4%) per month. Other categories were each selected by between 2.0% and 9.1% of respondents, including 10.6% who reported a monthly income of €4,000 or more.

Political affiliations were assessed by asking participants to indicate their preferred party (further explanation of political parties is provided in the supplementary materials). The results revealed a varied landscape: the largest share supported the Grüne (29.4%, n = 106), followed by the SPÖ (24.4%, n = 88) and NEOS (17.5%, n = 63). Smaller groups endorsed the KPÖ (7.5%, n = 27), ÖVP (6.7%, n = 24), and FPÖ (4.2%, n = 15). Minimal support was observed for Bier Die Bierpartei (1.9%, n = 7) and for GAZA and LMP, with each capturing 0.8% (n = 3). Only a few participants indicated voting for an “other party” (0.6%, n = 2) or selected “none of these” (1.4%, n = 5). Additionally, 2.5% (n = 9) stated they would not vote, and 2.2% (n = 8) would cast an invalid vote. This distribution underscores a politically diverse sample, with a clear lean toward green and social democratic ideologies compared to the recent popular vote results. Political parties were classified along the political spectrum (i.e., left- or right-wing) using the “smartmap,” a tool developed by the Department of Government of the University of Vienna [34].

For a full overview of our findings and an explanation of the political parties of Austria, see the supplementary table 3.

### Data Related to Usage of Conventional Preventive Health Services, Alternative or Complementary Methods

As described above, when we address CAM in this context, we refer to the intuitive understanding of it by participants after reading a brief clarification at the beginning of the survey, which can be found in the supplementary materials.

Over a duration of twelve months, 22.3% (n = 95) of respondents reported seeking alternative treatments, while 76.5% (n = 322) sought conventional care. When considering frequency, 8.4% (n = 36) had tried an alternative medicine treatment once, 9.6% (n = 41) used such treatments occasionally (several times per year), 2.3% (n = 10) did so regularly (monthly or more frequently), 11.7% (n = 50) used them rarely (once a year or less), and 68.0% (n = 291) had never used alternative treatments.

Regarding preventive health care, 79.9% (n = 342) had undergone preventive examinations in the past twelve months and intended to continue doing so, 1.4% (n = 6) had used them but did not plan to continue, 15.4% (n = 66) intended to use them in the future despite no prior experience, and 3.3% (n = 14) had neither used nor planned to use them.

Attitudes toward vaccination were predominantly positive: 74.7% (n = 316) of respondents fully agreed with the government’s current vaccination recommendations, 19.4% (n = 82) somewhat agreed, 3.3% (n = 14) somewhat disagreed, and 2.6% (n = 11) completely disagreed. Additionally, 49.6% (n = 207) of participants reported that they could explain the concept of evidence-based medicine.

### Data Related to Anthroposophic Medicine

With respect to anthroposophic medicine, 14.8% (n = 63) of respondents reported that they could explain the concept, 38.5% (n = 164) recognized the term without being able to fully define it, and 46.7% (n = 199) had never heard of it. When asked whether they viewed anthroposophy as scientific, 9.6% (n = 34) agreed, 32.0% (n = 114) partly agreed, 29.5% (n = 105) somewhat disagreed, and 28.9% (n = 103) totally disagreed. When asked whether doctors who are trained in AM are more competent than others, 11.1% (n = 40) agreed, 26.9% (n = 97) partly agreed, 24.1% (n = 87) somewhat disagreed, 38.0% (n = 137) totally disagreed that they are more competent.

We also found a very strong association (p < 0,001) indicating that individuals who had tried AM at least once were significantly more inclined to prefer CAM without conventional medicine in the hypothetical case vignette (mean scores: 55.68 ± 23.75 vs. 68.44 ± 22.98).

### Research questions & Regression Analysis

In evaluating healthcare utilization, subjects endorsing CAM, as they understood it, did not differ significantly in their use of preventive examinations (62.83 ± 26.28 vs. 62.08 ± 23.08; p = 0.792). In contrast, when examining attitudes toward vaccination, participants indicating a preference closer to exclusive CAM had a mean score of 46.68 ± 20.74, whereas those with higher scores (leaning toward conventional treatment) had a mean of 67.79 ± 22.24 (p < 0.001). This finding suggests that a preference for CAM (in the migraine example case) is associated with greater skepticism toward vaccinations.

The analyses yielded mixed results regarding the influence of chronic illnesses on the utilization of CAM services. Significant differences were observed for respiratory system comorbidities (p = 0.002), while other comorbidities (e.g., cancer, blood/immune system, nervous system) did not reach statistical significance. Furthermore, comparisons of perceived treatment within the healthcare system revealed no statistically significant differences (p = 0.174), although there was a trend toward lower perceived acknowledgement among those using CAM.

### Sociodemographic Factors

**Fig 3:**
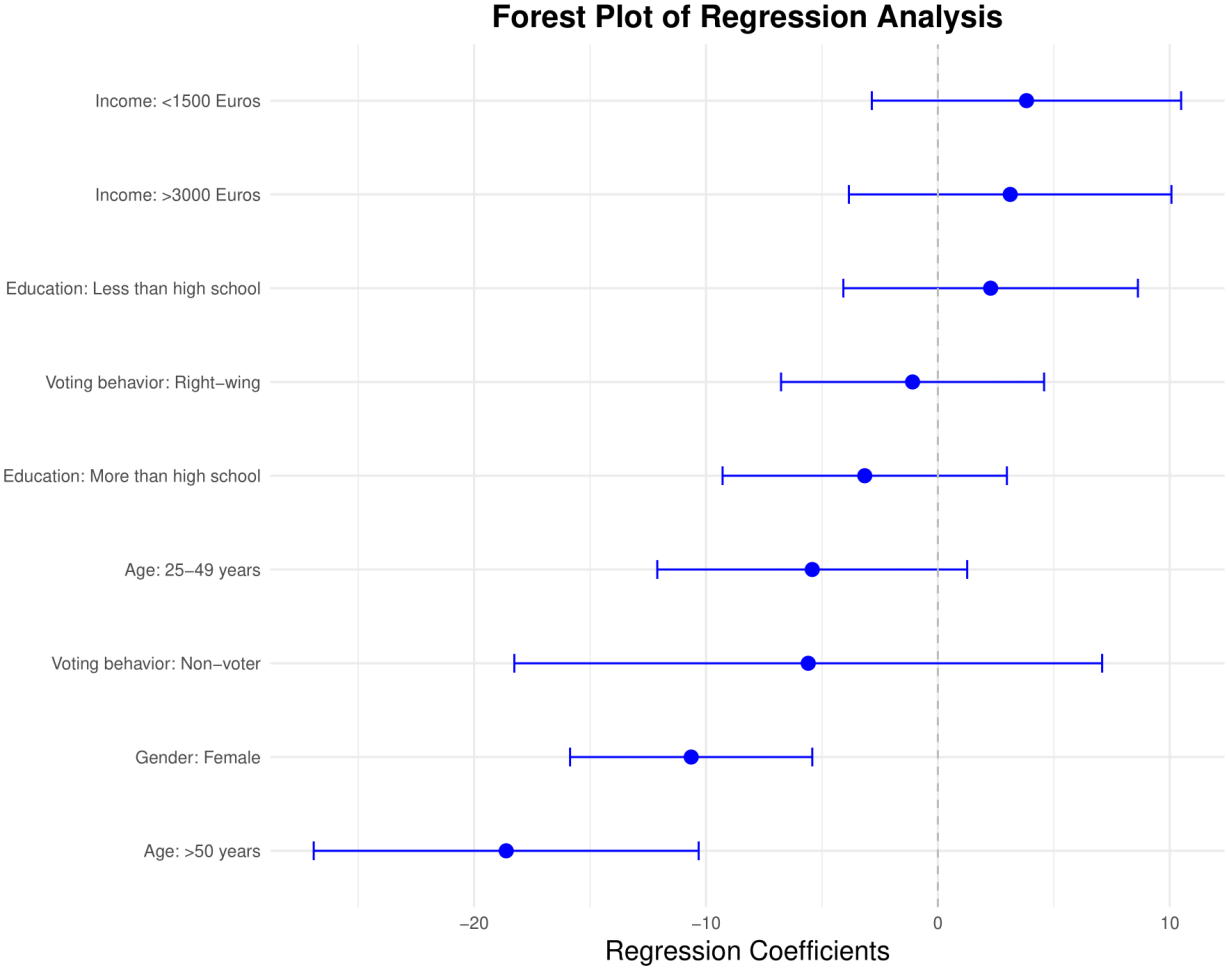
Regression Model of Sociodemographic Factors and Treatment Preference. For a table of the model, see supplementary materials.

A multiple regression analysis investigating the association between sociodemographic factors and treatment preference via example case revealed that individuals aged over 50 years had significantly lower scores (B = −18.62, SE = 4.24, t = −4.40, p < .001, 95% CI [−26.92, −10.32])compared to referebce age groups, indicating a stronger tendency toward CAM. Similarly, being female was associated with a lower score (B = −10.64, SE = 2.67, t = −3.99, p < .001, 95% CI [−15.86, −5.42]). In contrast, the age group 25–49 years (B = −5.42, SE = 3.41, t = −1.59, p = 0.113, 95% CI [−12.10, 1.26]) and variables such as education, income, and political orientation did not show statistically significant associations. The overall model explained 15.3% of the variance in treatment preference (R² = 0.153; Table 2, Fig 3).

**Table 2:** Unweighted multiple linear regression model predicting treatment preference (Fallvignette_2) from sociodemographic variables.

Predictor	B	SE	t	p	95% CI
Age 25–49 vs. 15–25	−5.42	3.41	−1.59	0.113	[-12.10, 1.26]
Age > 50 vs. 15–25	−18.62	4.24	−4.40	<0.001	[-26.92, -10.32]
Above Matura vs. Matura	−3.15	3.13	−1.01	0.314	[-9.29, 2.98]
Below Matura vs. Matura	2.27	3.24	0.70	0.483	[-4.08, 8.63]
Income >3000 € vs. 1500–3000 €	3.12	3.55	0.88	0.380	[-3.84, 10.07]
Income <1500 € vs. 1500–3000 €	3.82	3.40	1.12	0.262	[-2.85, 10.49]
Female vs. male	−10.64	2.67	−3.99	<0.001	[-15.86, -5.42]
Non-voter/ invalid vs. left	−5.59	6.47	−0.87	0.388	[-18.27, 7.08]
Right vs. left	−1.09	2.89	−0.38	0.706	[-6.76, 4.58]

Model fit: R² = 0.153, n = 336

We compared sociodemographic characteristics between participants who had tried AM at least once and those who had not. This analysis revealed a statistically significant difference in educational attainment (p < 0.001): individuals with an upper secondary school leaving certificate (Matura) were significantly less likely to have tried AM (27.6% among users versus 52.7% among non-users). In contrast, there was a tendency for both those with less than Matura-level education (43.9% vs. 24.6%) and those with higher education beyond Matura (28.6% vs. 22.7%) to have used AM. Additionally, female participants were significantly more inclined to have tried AM (p < 0.001). Although the associations with age (p = 0.083), residential setting (p = 0.035), and income (p = 0.092) did not reach conventional significance levels, there was a trend indicating that individuals over 50 years old, those earning more than €3000 per month, and residents of rural areas tended to use AM more frequently.

Furthermore, we examined respondents’ perceptions of AM as being scientific by contrasting those who viewed it as not scientific with those who considered it at least somewhat scientific. The unweighted analysis of sociodemographic characteristics revealed statistically significant differences in age (p < 0.001), residential area (p = 0.012), income (p < 0.001), and gender (p = 0.004). Notably, participants who viewed AM as somewhat scientific tended to be older, were more likely to live in rural areas, and included a higher proportion of women, whereas those who did not were predominantly younger and more urban. Although differences in educational attainment were observed (p = 0.112), they did not reach statistical significance.

### Attitudes toward science and anthroposophic medicine

Regarding the utilization of AM, several noteworthy differences emerged. While self-reported understanding of the term “evidence-based medicine” did not differ significantly between users and non-users (p = 0.430), significant group differences were observed in the evaluation of scientific literature. Specifically, proponents of AM placed greater value on long-established, “eminence-based” medical procedures (p = 0.001), whereas those more skeptical of AM placed greater importance on scientific evidence from clinical studies (p < 0.001). Furthermore, participants who considered AM to be scientifically valid demonstrated significantly lower levels of knowledge about evidence-based medicine (p < 0.001).

### Psychological predictors

A separate multiple regression model examining psychological variables as predictors of CAM utilization identified the following significant associations: (i) Perceived health control – higher levels were positively associated with greater CAM endorsement (B = 2.49, SE = 0.66, t = 3.76, p < .001, 95% CI [1.19, 3.79]). (ii) Prioritization of health optimization – a stronger focus on health optimization was significantly related to higher CAM endorsement (B = 1.78, SE = 0.83, t = 2.15, p = 0.033, 95% CI [0.15, 3.40]).

(iii) Ambiguity tolerance – lower tolerance for ambiguity was significantly associated with greater CAM endorsement (B = 1.66, SE = 0.66, t = 2.50, p = 0.013, 95% CI [0.36, 2.96]). (iv) Authoritarian attitudes – more pronounced authoritarian attitudes were also significantly associated with greater CAM endorsement (B = 1.72, SE = 0.71, t = 2.43, p = 0.015, 95% CI [0.33, 3.10])..

**Fig 4:**
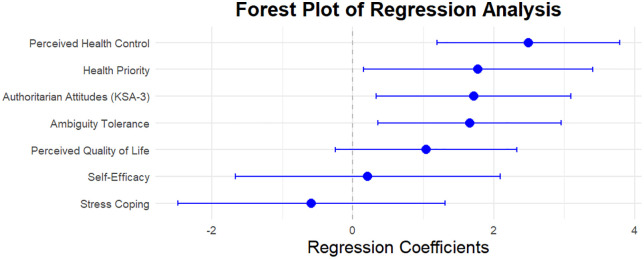
Regression Model of Psychological Predictors for Endorsement of Alternative and Complementary Medicine. For a table of the model, see supplementary table 4.

In contrast, perceived quality of life (B = 1.04, SE = 0.66, t = 1.59, p = 0.114, 95% CI [−0.25, 2.33]), stress management (B = −0.59, SE = 0.97, t = −0.61, p = 0.544, 95% CI [−2.48, 1.31]), and self-efficacy (B = 0.21, SE = 0.96, t = 0.22, p = 0.826, 95% CI [−1.67, 2.09]) did not show statistically significant associations. The overall model explained 13.7% of the variance in treatment preference (R² = 0.137; Table 3, Fig 4).

**Table 3:** Unweighted multiple linear regression model predicting treatment preference (Fallvignette_2_gedreht) from psychological variables.

Predictor	B	SE	t	p	95% CI
Perceived quality of life	1.04	0.66	1.59	0.114	[-0.25, 2.33]
Perceived health control	2.49	0.66	3.76	<0.001	[1.19, 3.79]
Stress coping	−0.59	0.97	−0.61	0.544	[-2.48, 1.31]
Self-efficacy	0.21	0.96	0.22	0.826	[-1.67, 2.09]
Health priority	1.78	0.83	2.15	0.033	[0.15, 3.40]
Ambiguity tolerance	1.66	0.66	2.50	0.013	[0.36, 2.96]
Authoritarian attitudes	1.72	0.71	2.43	0.015	[0.33, 3.10]

Model fit: R² = 0.137, n = 362.

### Regression Models Post-Stratified

**Fig 5:**
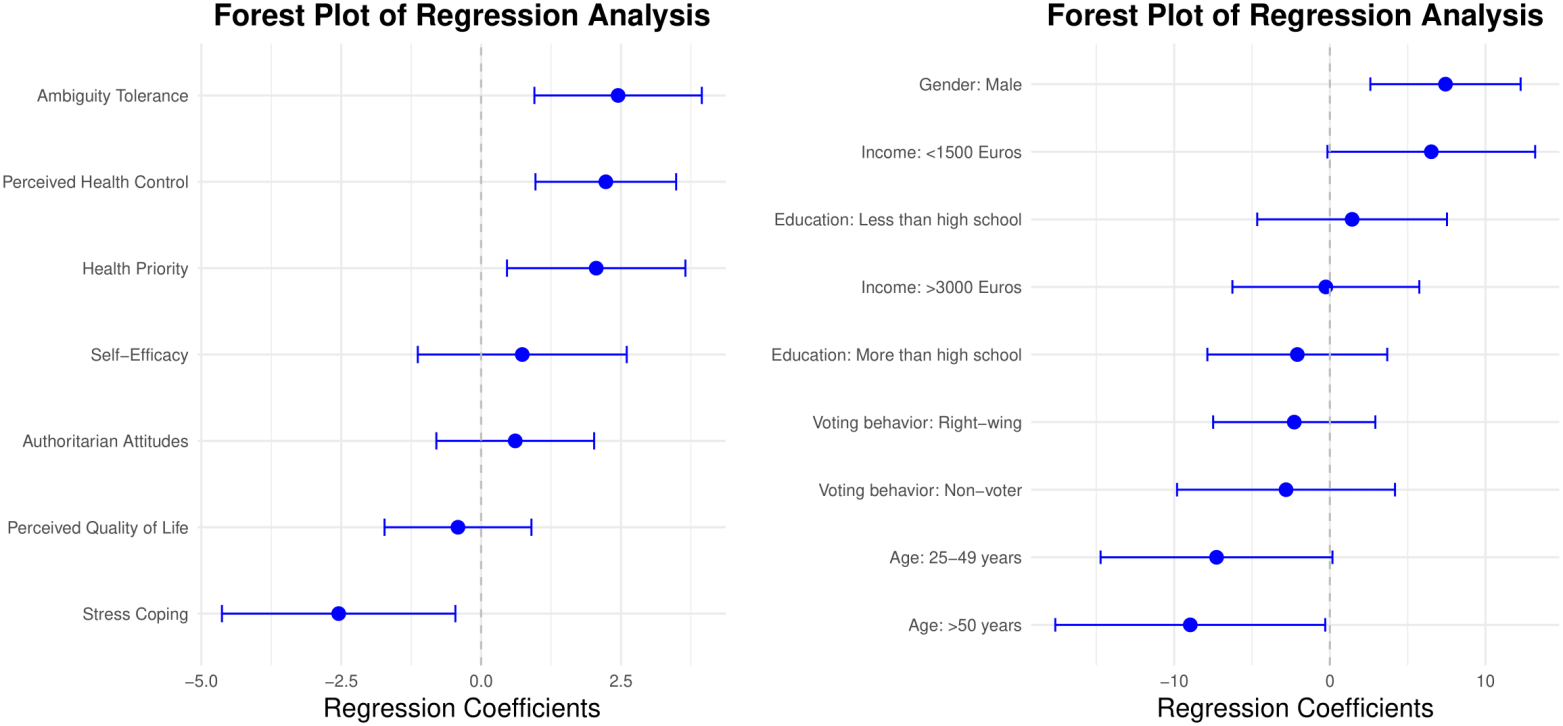
Post-Stratified Regression Models of Sociodemographic and Psychological Predictors of Treatment Preferences. For a table of the values underlying the model, see supplementary S1-S2 File.

In the primary regression model, age and gender were significant predictors of treatment preference. Individuals aged 25–49 and those over 50 scored lower than the reference group (B = −5.96, SE = 3.95, t = −1.51, p = 0.132, 95% CI [−13.71, 1.78] and B = −14.86, SE = 4.70, t = −3.16, p = 0.002, 95% CI [−24.06, −5.65], respectively), while male gender was associated with higher scores than female gender (B = 11.61, SE = 2.53, t = 4.60, p < .001, 95% CI [6.66, 16.56]). Education and income per month did not show significant effects overall, although above Matura approached significance (B = −6.11, SE = 3.30, t = −1.85, p = 0.065, 95% CI [−12.59, 0.36]). Lower income (<1500 Euro) was associated with higher scores than the reference group (B = 7.54, SE = 3.76, t = 2.00, p = 0.046, 95% CI [0.17, 14.91]). The weighted sociodemographic model explained 23.7% of the variance in treatment preference (R² = 0.237; Table 4, Fig 5).

**Table 4:** Weighted multiple linear regression model predicting treatment preference (Fallvignette_2) from sociodemographic variables.

Predictor	B	SE	t	p	95% CI
Age 25–49 vs. 15–25	−5.96	3.95	−1.51	0.132	[-13.71, 1.78]
Age > 50 vs. 15–25	−14.86	4.70	−3.16	0.002	[-24.06, -5.65]
Above Matura vs. Matura	−6.11	3.30	−1.85	0.065	[-12.59, 0.36]
Below Matura vs. Matura	1.83	3.06	0.60	0.551	[-4.17, 7.83]
Income >3000 € vs. 1500–3000 €	−1.51	3.19	−0.47	0.637	[-7.76, 4.75]
Income <1500 € vs. 1500–3000 €	7.54	3.76	2.00	0.046	[0.17, 14.91]
Male vs. female	11.61	2.53	4.60	<0.001	[6.66, 16.56]
Non-voter/ invalid vs. left	−6.63	4.14	−1.60	0.111	[-14.75, 1.49]
Right vs. left	−1.24	2.73	−0.45	0.650	[-6.59, 4.11]

Model fit: R² = 0.237, n = 334.

In the weighted psychological model, self-efficacy emerged as the strongest positive predictor of CAM preference (B = 4.07, SE = 1.08, t = 3.75, p < .001, 95% CI [1.94, 6.19]). In addition, prioritization of health (B = 2.34, SE = 0.90, t = 2.61, p = 0.010, 95% CI [0.58, 4.09]), and ambiguity tolerance (B = 1.82, SE = 0.77, t = 2.36, p = 0.019, 95% CI [0.31, 3.34]) were all positively associated with a tendency to choose only CAM treatment. Perceived health control showed only a trend-level association (B = 1.30, SE = 0.76, t = 1.71, p = 0.089, 95% CI [−0.19, 2.78]). Perceived quality of life (B = −0.29, SE = 0.72, t = −0.41, p = 0.686, 95% CI [−1.71, 1.12]), stress coping (B = −1.80, SE = 1.13, t = −1.59, p = 0.114, 95% CI [−4.02, 0.42]), and authoritarian attitudes (B = 0.26, SE = 0.80, t = 0.33, p = 0.745, 95% CI [−1.31, 1.83]) were not statistically significant. The weighted psychological model explained 14.6% of the variance in treatment preference (R² = 0.146; Table 5).

**Table 5:** Weighted multiple linear regression model predicting treatment preference from psychological variables.

Predictor	B	SE	t	p	95% CI
Perceived quality of life	−0.29	0.72	−0.41	0.686	[-1.71, 1.12]
Perceived health control	1.30	0.76	1.71	0.089	[-0.19, 2.78]
Stress coping	−1.80	1.13	−1.59	0.114	[-4.02, 0.42]
Self-efficacy	4.07	1.08	3.75	<0.001	[1.94, 6.19]
Health priority	2.34	0.90	2.61	0.010	[0.58, 4.09]
Ambiguity tolerance	1.82	0.77	2.36	0.019	[0.31, 3.34]
Authoritarian attitudes	0.26	0.80	0.33	0.745	[-1.31, 1.83]

Model fit: R² = 0.146, n = 305.

In summary, after post-stratification, the associations between sociodemographic characteristics and treatment preference remained consistent, with female gender and age over 50 continuing to predict a stronger inclination toward CAM. In contrast, the pattern of psychological predictors was notably altered. Specifically, authoritarian attitudes were no longer significantly associated with CAM preference, whereas high stress coping ability emerged as a significant negative predictor of CAM endorsement.

### Combined Authoritarian and Ambiguity-Tolerance Related Hypotheses

Group comparisons based on the frequency of agreement responses in the psychological section of the survey—focusing on ambiguity tolerance and authoritarian orientation (as measured by the KSA-3)—revealed several remarkable findings. Importantly, these results remained robust after post-stratification.

**Fig 6:**
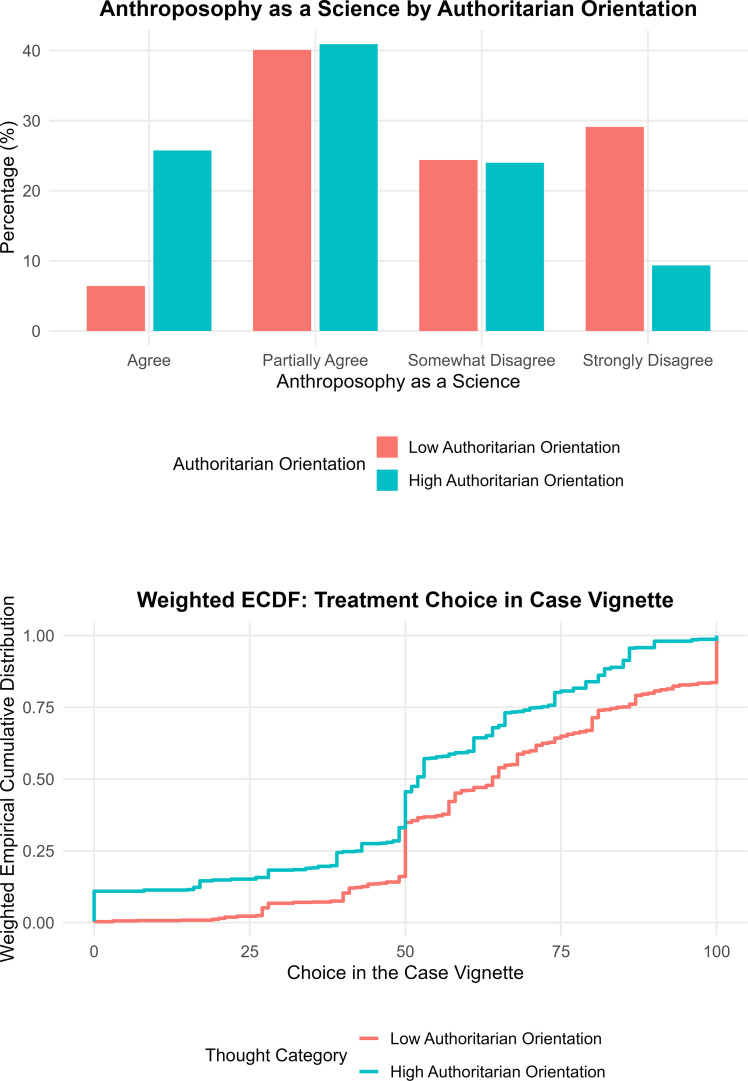
Post-stratified data: (1) Belief that anthroposophic medicine is scientific among participants with low vs. high authoritarian orientation. (2) ECDF-Plot (Empirical Cumulative Distribution Function) illustrates the response distribution for a metric variable capturing the tendency to cho‌‌ose CAM, conventional medicine, or both. A comparison with pre-stratified graphs can be found in the supplementary materials (supplementary figure 5).

Participants with higher authoritarian orientation (characterized by low ambiguity tolerance and strong authoritarian leanings) reported significantly higher spending on CAM (p = 0.004). While no significant difference was observed in familiarity with AM (p = 0.363), those with higher authoritarian orientation were significantly more likely to rate AM as scientifically valid (p < 0.001). Interestingly, despite this perception, there was only a marginal trend toward having used AM at least once (p = 0.536). Authoritarian orientation was also significantly associated with a greater likelihood of selecting CAM-only approaches in the hypothetical case vignette (p < 0.001), with a stronger preference for eminence-based over evidence-based approaches (p < 0.001). Finally, a significant association was found between specific political party preferences and authoritarian orientation (p < 0.001; Fig 6).

## Discussion

To better understand the continued relevance of AM, it is essential to address questions of scientific validity and examine the broader political, cultural, and psychological contexts in which it persists. This socio-psychological study explores the social and individual conditions contributing to anthroposophic healthcare’s appeal and uptake in contemporary Austria.

Rather than evaluating the empirical foundations of AM, the focus lies on examining what beliefs, values, and sociopolitical factors shape engagement with it, and how patterns of perception and behavior relate to its cultural resilience.

This is the first survey in Austria – or any German-speaking country – to systematically examine beliefs about AM alongside sociodemographic, political, and psychological variables.. This is particularly relevant given that both anthroposophy and its associated medical system originated in this region. Our findings reveal a spectrum of awareness regarding AM. A significant portion of respondents (46.7%) reported a complete lack of familiarity with it. This is a noteworthy observation, especially given that a substantial 41% of participants partly agreed that AM is scientific. This contrast highlights that both a lack of awareness and a perception of scientific credibility exist within our study population, setting the stage for further investigation into the impact of demographic characteristics and underlying psychological traits on the uptake of CAM and AM. In line with previous research, our findings confirm that individuals identifying as female and those over the age of 50 are more likely to engage with CAMEducational attainment played a notable role: participants holding an upper secondary school leaving certificate (Matura) were significantly less likely to have used AM compared to those with either lower or higher levels of education. These patterns are broadly consistent with established findings in the CAM literature, where female gender, older age, and higher income have been repeatedly identified as predictors of CAM use across European populations [35].

Female participants consistently showed a higher likelihood of both using AM and perceiving it as scientific. Additional trends emerged in age, residential setting, and income, with older individuals, rural residents, and those with higher incomes more likely to use AM and consider it scientifically legitimate.

Psychologically, a lower tolerance for ambiguity in expert opinions and a stronger belief in personal responsibility for health—rather than viewing it as a matter of luck—also distinguished those who endorsed AM. Taken together, these findings highlight a complex interplay of sociodemographic and psychological factors that shape engagement with and perceptions of AM.

While the relationship between esoteric elements of AM and authoritarian psychological structures has been a topic of theoretical discussion for nearly a century [23], empirical evidence has remained largely elusive. Our findings help to bridge this gap by demonstrating a clear correlation between a belief that AM is scientific and a tendency toward these psychological dispositions. In the unweighted model, low ambiguity tolerance (B = 1.66, p = .013), prioritization of health optimization (B = 1.78, p < .001), and authoritarian attitudes (B = 1.72, p = .015) were each significantly associated with greater CAM endorsement.

Importantly, post-stratified regression analyses yielded insights that both reinforce and refine our initial findings. While the associations between sociodemographic factors and treatment preferences remained robust—confirming that female gender and being over the age of 50 consistently predict a greater propensity toward CAM—the pattern of psychological predictors shifted. Initially, authoritarian attitudes were significantly associated with a preference for CAM; however, following post-stratification, this association was no longer significant, whereas low ambiguity tolerance retained its robust significance. Additionally, high stress coping ability—defined as a low tendency to trivialize or respond inadequately to high stress—emerged as a significant negative predictor of CAM endorsement.

These findings suggest that individuals with lower stress tolerance are more likely to prefer CAM modalities. Several mechanisms may account for this association. CAM practices typically involve longer consultations and a more participatory therapeutic relationship, which may offer patients a greater sense of agency in treatment decisions. This may be particularly appealing for individuals who experience conventional medical encounters as anxiety-inducing. The emphasis in many CAM modalities on holistic framing and relational care may also reduce situational distress. Conversely, conventional medicine’s reliance on probabilistic reasoning, risk communication, and standardized protocols may be particularly aversive for individuals with low stress tolerance. These interpretations remain speculative and would require targeted investigation through qualitative or mixed-methods designs. In the post-stratified analysis, authoritarian attitudes were no longer significantly associated with CAM preference. Several factors may contribute to this shift. First, post-stratification by political party affiliation may absorb shared variance, given the well-documented correlation between authoritarianism and political orientation. Second, the KSA-3 is a brief screening instrument that may lack the sensitivity required to detect effects in non-clinical populations. Third, the construct of authoritarianism itself (as originally operationalized by Adorno and Frenkel-Brunswik [36]) may require updated measurement approaches to capture its contemporary manifestations, which may be less overtly ideological and more diffusely epistemic. Importantly, the non-significance pertains specifically to CAM *preference*; the association between authoritarian orientation and the *perceived scientific validity* of AM remained robust (p < .001), suggesting that authoritarian dispositions may shape epistemic judgments more strongly than behavioral choices. By integrating measures of ambiguity tolerance and authoritarian predisposition, our analysis revisits longstanding theoretical propositions that posit a close connection between authoritarian personality and esoteric beliefs, as exemplified in anthroposophy and AM. Drawing on the foundational work of Adorno and Frenkel-Brunswik [36]—who conceptualized the authoritarian personality and identified low ambiguity tolerance as a core component—our evaluation demonstrates that individuals with heightened authoritarian tendencies are more inclined to invest in CAM treatments. In fact, our results show a statistically significant association (p < 0.001) between authoritarian orientation defined this way and the conviction that AM is scientifically valid. An observation that becomes even more fascinating when contrasting it with the finding that there is no statistically significant correlation between authoritarian orientation and whether a participant has tried AM before – only a slight tendency (p = 0.536). This dissociation between the robust link to epistemic judgment and the absent link to behavior merits closer attention. It suggests that authoritarian dispositions operate primarily at the level of knowledge evaluation rather than treatment choice: individuals with higher authoritarian orientation are not more likely to have used AM, but they are significantly more likely to regard it as scientifically valid. This pattern is consistent with theoretical accounts of authoritarianism as a disposition toward deference to perceived legitimate authority [36]. Within this framework, “scientific validity” may function less as an empirical assessment and more as a marker of epistemic trust: a judgment shaped by the perceived authority of the source rather than by engagement with the underlying evidence. The finding thus opens a productive line of inquiry into how the category of “scientific validity” itself operates as a site of authority attribution, and how psychological dispositions mediate the reception of epistemic claims in healthcare contexts.

Furthermore, the observed correlations with political party affiliation suggest that authoritarian personality traits extend beyond health-related behaviors, bearing considerable political implications. This dynamic is further underscored by the finding that a preference for CAM treatment (in the migraine example vignette) is associated with greater skepticism toward vaccinations (<0.001): a highly politicized topic nowadays [37–39].

These findings point beyond the immediate question of CAM use to the broader relationship between epistemic authority and psychological disposition. As Adorno argued, ideology functions as justification [40]: the mechanisms by which epistemic claims acquire or lose legitimacy are inseparable from the cultural and psychological conditions in which they are evaluated. Effective science communication, accordingly, cannot rely solely on disseminating evidence but must also address the dispositional and structural conditions that shape its reception.

The present study employs empirical methods to investigate the psychological conditions under which epistemic authority is granted or withheld: standardized instruments, regression models, and significance testing. In doing so, it operates within the same regime of epistemic authority whose dynamics it seeks to examine. This is not a contradiction to be resolved but a tension to be acknowledged. Our study occupies a position that is simultaneously within and against the epistemological structures it interrogates. The tools of empirical social science do not merely describe epistemic phenomena; they participate in constituting “scientific validity” as a normative category. As the sociology of scientific knowledge has demonstrated, the criteria by which claims are admitted as legitimate knowledge are themselves products of historically contingent social practices, not reflections of a neutral epistemological order [41]. The symmetry principle applies here reflexively: the same analytical resources that explain why respondents with authoritarian dispositions grant epistemic authority to AM must, in principle, apply to the epistemic authority claimed by our own instruments and inferential procedures. There is no vantage point exterior to this entanglement, no metalanguage from which the construction of validity could be observed without simultaneously being performed. Acknowledging this does not invalidate the findings. It does, however, resist the assumption that positivist methodology occupies a privileged position outside the epistemic dynamics it purports to describe, and it situates the present study as an intervention within, rather than an adjudication upon, the field of competing knowledge claims it investigates.

### Strengths and Weaknesses

As noted above, a diverse set of data was collected from participants, enabling the examination of associations among various socioeconomic, political, and psychological factors. Additionally, this study applied a novel scientific perspective to an area that has not previously been examined in this manner. Although AM and its contested status as a scientific discipline have been widely debated, empirical investigations and substantial data remain scarce.

An important limitation of the study is that we can only report correlations and, while it might be tempting to interpret and hypothesize causal relationships, our data simply do not allow this. Furthermore, participants were recruited using an online convenience sampling approach, which introduces the possibility of selection bias. Individuals who chose to complete the questionnaires may systematically from those who did not. In particular, it is possible, that persons with a stronger interest in the CAM were more likely to respond. Therefore, the sample cannot be considered representative of the Austrian general population.

The study sample also exhibited systematic biases attributable to the method of survey distribution, via social media groups and direct messages, which led to an overrepresentation of individuals with left-leaning political orientations, particularly those affiliated with the Green Party and Social Democratic parties. To partially address these distributional imbalances, post-stratification weighting based on Austrian National Council election results was applied in the regression analyses. However, this procedure can only adjust for known imbalances and does not fully eliminate potential sampling bias.

Another methodological limitation is that the study was not preregistered and no formal a priori power analysis was conducted. As a result, the analyses should be interpreted as exploratory rather than confirmatory.

Several abbreviated or adapted multi-item measures showed only low to moderate internal consistency, which may have attenuated associations and limits the interpretability of findings involving these constructs.

## Conclusion

Our analyses suggest that ideological predispositions, such as ambiguity tolerance and authoritarian attitudes, are associated with the perception of AM as scientifically valid. Moreover, we replicated established socioeconomic patterns — namely, the higher prevalence of CAM use among individuals identifying as female and those over the age of 50—along with the significant role of perceived control over one’s health in predicting non-conventional medical preferences, underscores the complex interplay between individual beliefs and broader cultural narratives.

While these findings are intriguing, we remain cautious about making broad generalizations. They underscore the promise of future studies that integrate both qualitative and quantitative approaches to more effectively elucidate the underlying mechanisms. Moreover, our study demonstrates how such integration may be achieved through a highly interdisciplinary approach: incorporating insights and methodologies from history, sociology, psychology, and philosophy.

## Supporting information

S1 Table All measures stratified by gender.
(PDF)

S2 Table All measures stratified by leaning towards conventional vs. CAM.
(PDF)

S3 Table Regression model of sociodemographic factors.
(PDF)

S4 Table Regression model of psychological predictors.
(PDF)

S1 Fig Differences of pre- and post-stratified data in authoritarian orientation.
(PDF)

S1 File Survey questions.
(PDF)

S2 File Explanation of political parties.
(PDF)
