# Supplementary material for: Authoritarian attitudes and the perceived scientific legitimacy of anthroposophic medicine: A survey of attitudes on complementary and alternative medicine in Austria
Source: PLoS One. 2026 Jun 17;21(6):e0348672. doi: 10.1371/journal.pone.0348672 (PMC13274894; doi:10.1371/journal.pone.0348672)
Supplement: S1 Table — All measures stratified by gender. (PDF) [file pone.0348672.s001.pdf]

Supplement 1: All measures stratified by gender

Variables	Level	Overall	Male	Female	p
n		429	143	286	
Satisfaction with conventional medicine (mean (SD))		2.00 (0.05)	1.99 (0.08)	2.00 (0.00)	0.155
Satisfaction with alternative medicine (mean (SD))		1.50 (0.50)	1.31 (0.47)	1.59 (0.49)	<0.001
Satisfaction with complementary medicine (mean (SD))		1.61 (0.49)	1.46 (0.50)	1.69 (0.46)	<0.001
Overall life satisfaction (mean (SD))		3.71 (0.71)	3.76 (0.74)	3.68 (0.69)	0.325
Positive outlook for the future (mean (SD))		3.39 (0.88)	3.40 (0.92)	3.39 (0.87)	0.946
Regular worries in life (mean (SD))		3.10 (1.03)	2.91 (1.01)	3.19 (1.03)	0.008
Spiritual beliefs help with difficulties (mean (SD))		2.68 (1.27)	2.58 (1.32)	2.73 (1.24)	0.262
Mean (±SD) response score: Average rating for a migraine case scenario on a 0–100 scale (0 = conventional medicine; 100 = CAM), with variability indicated by the standard deviation.		62.23 (23.73)	68.76 (26.38)	58.96 (21.61)	<0.001
Perceived control over health (%)	1	0 (0)	0 (0)	0 (0)	0.125
	2	27 (6.4)	8 (5.9)	19 (6.7)	
	3	65 (15.5)	13 (9.6)	52 (18.3)	
	4	255 (60.7)	90 (66.2)	165 (58.1)	
	5	73 (17.4)	25 (18.4)	48 (16.9)	
Illness perceived as a matter of luck (rather than misfortune) (%)	1	66 (15.8)	18 (13.1)	48 (17.0)	0.086
	2	164 (39.1)	62 (45.3)	102 (36.2)	
	3	85 (20.3)	32 (23.4)	53 (18.8)	
	4	93 (22.2)	21 (15.3)	72 (25.5)	
	5	11 (2.6)	4 (2.9)	7 (2.5)	
Tendency to reassure oneself and accept the situation rather than acting (%)	1	13 (3.1)	5 (3.7)	8 (2.8)	0.897
	2	64 (15.3)	21 (15.4)	43 (15.3)	
	3	91 (21.8)	26 (19.1)	65 (23.1)	
	4	189 (45.3)	63 (46.3)	126 (44.8)	
	5	60 (14.4)	21 (15.4)	39 (13.9)	

Variables	Level	Overall	Male	Female	p
Active approach to resolving the cause of distress instead of just self-soothing (%)	1	0 (0)	0 (0)	0 (0)	0.285
	2	24 (5.7)	11 (8.0)	13 (4.6)	
	3	45 (10.7)	14 (10.2)	31 (10.9)	
	4	231 (54.9)	68 (49.6)	163 (57.4)	
	5	121 (28.7)	44 (32.1)	77 (27.1)	
Self-blame for illness (%)	1	61 (14.5)	18 (13.0)	43 (15.2)	0.920
	2	186 (44.2)	61 (44.2)	125 (44.2)	
	3	100 (23.8)	36 (26.1)	64 (22.6)	
	4	70 (16.6)	22 (15.9)	48 (17.0)	
	5	4 (1.0)	1 (0.7)	3 (1.1)	
Every problem has a solution (%)	1	13 (3.1)	0 (0.0)	13 (4.6)	0.021
	2	43 (10.2)	9 (6.5)	34 (12.0)	
	3	55 (13.1)	16 (11.6)	39 (13.8)	
	4	239 (56.8)	89 (64.5)	150 (53.0)	
	5	71 (16.9)	24 (17.4)	47 (16.6)	
Ability to cope well with events (%)	1	4 (1.0)	1 (0.7)	3 (1.1)	0.061
	2	27 (6.4)	4 (2.9)	23 (8.1)	
	3	68 (16.2)	23 (16.8)	45 (15.8)	
	4	242 (57.5)	74 (54.0)	168 (59.2)	
	5	80 (19.0)	35 (25.5)	45 (15.8)	
Adjusting daily life for health (%)	1	2 (0.5)	0 (0.0)	2 (0.7)	0.013
	2	26 (6.2)	13 (9.6)	13 (4.6)	
	3	40 (9.6)	12 (8.8)	28 (10.0)	
	4	214 (51.3)	80 (58.8)	134 (47.7)	
	5	135 (32.4)	31 (22.8)	104 (37.0)	
Refraining from indulgences for health (%)	1	3 (0.7)	1 (0.7)	2 (0.7)	0.027
	2	42 (10.1)	23 (16.9)	19 (6.8)	
	3	40 (9.6)	11 (8.1)	29 (10.4)	
	4	204 (49.0)	65 (47.8)	139 (49.6)	
	5	127 (30.5)	36 (26.5)	91 (32.5)	

Variables	Level	Overall	Male	Female	p
Endorsing the notion that sharing similar values is beneficial (%)	1	55 (14.3)	27 (21.4)	28 (10.9)	0.086
	2	66 (17.2)	18 (14.3)	48 (18.6)	
	3	76 (19.8)	24 (19.0)	52 (20.2)	
	4	118 (30.7)	37 (29.4)	81 (31.4)	
	5	69 (18.0)	20 (15.9)	49 (19.0)	
Belief that experts who cannot give clear answers likely lack knowledge (%)	1	151 (36.8)	62 (45.9)	89 (32.4)	0.005
	2	130 (31.7)	41 (30.4)	89 (32.4)	
	3	62 (15.1)	11 (8.1)	51 (18.5)	
	4	54 (13.2)	14 (10.4)	40 (14.5)	
	5	13 (3.2)	7 (5.2)	6 (2.2)	
Preference for strong leaders (%)	1	76 (18.6)	30 (22.2)	46 (16.8)	0.090
	2	103 (25.2)	41 (30.4)	62 (22.6)	
	3	87 (21.3)	28 (20.7)	59 (21.5)	
	4	117 (28.6)	28 (20.7)	89 (32.5)	
	5	26 (6.4)	8 (5.9)	18 (6.6)	
Not questioning tried-and-tested behaviours (%)	1	149 (36.1)	52 (39.4)	97 (34.5)	0.388
	2	145 (35.1)	38 (28.8)	107 (38.1)	
	3	58 (14.0)	22 (16.7)	36 (12.8)	
	4	54 (13.1)	17 (12.9)	37 (13.2)	
	5	7 (1.7)	3 (2.3)	4 (1.4)	
Grouped by rating for a migraine case scenario on a 0–100 scale (0 = conventional medicine; 100 = CAM), with variability indicated by the standard deviation	Favours conventional	282 (65.7)	106 (74.1)	176 (61.5)	0.013
	Favours alternative	147 (34.3)	37 (25.9)	110 (38.5)	
Number of comorbidities (mean (SD)		1.94 (1.45)	1.74 (1.35)	2.05 (1.49)	0.041
Comorbidities (%)	FALSE	103 (24.6)	38 (27.1)	65 (23.3)	0.458
	TRUE	316 (75.4)	102 (72.9)	214 (76.7)	
Recent infections (%)	Checked	194 (45.2)	65 (45.5)	129 (45.1)	1.000
	Not checked	235 (54.8)	78 (54.5)	157 (54.9)	
Comorbidity: Cancer (%)	Checked	9 (2.1)	3 (2.1)	6 (2.1)	1.000

Variables	Level	Overall	Male	Female	p
	Not checked	420 (97.9)	140 (97.9)	280 (97.9)	
Blood/immune system conditions (%)	Checked	16 (3.7)	2 (1.4)	14 (4.9)	0.126
	Not checked	413 (96.3)	141 (98.6)	272 (95.1)	
Nutritional/metabolic diseases (%)	Checked	33 (7.7)	9 (6.3)	24 (8.4)	0.564
	Not checked	396 (92.3)	134 (93.7)	262 (91.6)	
Nervous system conditions (%)	Checked	11 (2.6)	4 (2.8)	7 (2.4)	1.000
	Not checked	418 (97.4)	139 (97.2)	279 (97.6)	
Psychological conditions (%)	Checked	74 (17.2)	14 (9.8)	60 (21.0)	0.006
	Not checked	355 (82.8)	129 (90.2)	226 (79.0)	
Eye conditions (%)	Checked	26 (6.1)	10 (7.0)	16 (5.6)	0.721
	Not checked	403 (93.9)	133 (93.0)	270 (94.4)	
Ear conditions (%)	Checked	21 (4.9)	11 (7.7)	10 (3.5)	0.097
	Not checked	408 (95.1)	132 (92.3)	276 (96.5)	
Cardiovascular conditions (%)	Checked	20 (4.7)	10 (7.0)	10 (3.5)	0.169
	Not checked	409 (95.3)	133 (93.0)	276 (96.5)	
Respiratory conditions (%)	Checked	46 (10.7)	13 (9.1)	33 (11.5)	0.544
	Not checked	383 (89.3)	130 (90.9)	253 (88.5)	
Digestive system conditions (%)	Checked	53 (12.4)	14 (9.8)	39 (13.6)	0.324
	Not checked	376 (87.6)	129 (90.2)	247 (86.4)	
Skin conditions (%)	Checked	56 (13.1)	19 (13.3)	37 (12.9)	1.000
	Not checked	373 (86.9)	124 (86.7)	249 (87.1)	
Musculoskeletal conditions (%)	Checked	55 (12.8)	10 (7.0)	45 (15.7)	0.016
	Not checked	374 (87.2)	133 (93.0)	241 (84.3)	
Urogenital conditions (%)	Checked	36 (8.4)	4 (2.8)	32 (11.2)	0.006
	Not checked	393 (91.6)	139 (97.2)	254 (88.8)	
Congenital malformations (%)	Checked	6 (1.4)	1 (0.7)	5 (1.7)	0.663
	Not checked	423 (98.6)	142 (99.3)	281 (98.3)	
Injuries (%)	Checked	79 (18.4)	30 (21.0)	49 (17.1)	0.403
	Not checked	350 (81.6)	113 (79.0)	237 (82.9)	
No comorbidities (%)	Checked	74 (17.2)	23 (16.1)	51 (17.8)	0.752
	Not checked	355 (82.8)	120 (83.9)	235 (82.2)	

Variables	Level	Overall	Male	Female	p
Prefer not to answer – Comorbidities (%)	Checked	10 (2.3)	3 (2.1)	7 (2.4)	1.000
	Not checked	419 (97.7)	140 (97.9)	279 (97.6)	
Other diseases (%)	Checked	17 (4.0)	5 (3.5)	12 (4.2)	0.930
	Not checked	412 (96.0)	138 (96.5)	274 (95.8)	
Taken seriously by healthcare professionals (%)	I was almost always taken seriously	139 (32.8)	65 (46.8)	74 (26.0)	<0.001
	I was mostly taken seriously	146 (34.4)	46 (33.1)	100 (35.1)	
	Half and half	124 (29.2)	24 (17.3)	100 (35.1)	
	I was almost never taken seriously	2 (0.5)	1 (0.7)	1 (0.4)	
	I was mostly NOT taken seriously	13 (3.1)	3 (2.2)	10 (3.5)	
Taken seriously by healthcare professionals - Different grouping (%)	I was mostly taken seriously	146 (34.0)	46 (32.2)	100 (35.0)	<0.001
	Some to little	144 (33.6)	32 (22.4)	112 (39.2)	
	I was almost always taken seriously	139 (32.4)	65 (45.5)	74 (25.9)	
Money spent on evidence-based medicine (%)	0€	123 (29.4)	56 (40.3)	67 (24.0)	0.010
	1-100€	36 (8.6)	15 (10.8)	21 (7.5)	
	100-250€	85 (20.3)	26 (18.7)	59 (21.1)	
	250-500€	78 (18.7)	18 (12.9)	60 (21.5)	
	500-750€	47 (11.2)	11 (7.9)	36 (12.9)	
	750-1000€	23 (5.5)	5 (3.6)	18 (6.5)	
	1000€ or more	26 (6.2)	8 (5.8)	18 (6.5)	
Money spent on eminence-based medicine (%)	0€	276 (65.6)	109 (77.9)	167 (59.4)	0.015
	1-100€	48 (11.4)	8 (5.7)	40 (14.2)	
	100-250€	38 (9.0)	9 (6.4)	29 (10.3)	
	250-500€	27 (6.4)	5 (3.6)	22 (7.8)	
	500-750€	15 (3.6)	5 (3.6)	10 (3.6)	
	750-1000€	8 (1.9)	2 (1.4)	6 (2.1)	
	1000€ or more	9 (2.1)	2 (1.4)	7 (2.5)	

Variables	Level	Overall	Male	Female	p
Location (%)	Countryside	123 (30.5)	36 (27.1)	87 (32.2)	0.346
	City	280 (69.5)	97 (72.9)	183 (67.8)	
Sports (%)	No sporting activity	36 (8.6)	12 (8.6)	24 (8.6)	0.105
	Less than 1 hour per week	57 (13.6)	17 (12.2)	40 (14.3)	
	Regularly, 1–2 hours per week	126 (30.1)	36 (25.9)	90 (32.1)	
	Regularly, 2–4 hours per week	122 (29.1)	38 (27.3)	84 (30.0)	
	Regularly, more than 4 hours per week	78 (18.6)	36 (25.9)	42 (15.0)	
Nutrition (%)	Predominantly plant-based and freshly cooked meals, legumes (e.g., lentils, beans), lots of fruits and vegetables, whole grain products, fish, healthy fats (e.g., nuts, avocado), hardly any snacks or finger foods, hardly any sweet baked goods.	91 (21.4)	33 (23.4)	58 (20.4)	0.057
	Regularly plant-based and freshly cooked meals, legumes (e.g., lentils, beans), often fruits and vegetables, whole grain products, fish, healthy fats (e.g., nuts, avocado), occasionally snacks and finger foods, occasionally sweet baked goods.	279 (65.6)	82 (58.2)	197 (69.4)	
	Often fast food, snacks and finger foods, sweet baked goods, processed	47 (11.1)	23 (16.3)	24 (8.5)	

Variables	Level	Overall	Male	Female	p
	meat products (e.g., sausage), red meat, full-fat dairy products, sweetened beverages, sometimes freshly cooked meals, occasionally fruits and vegetables.				
	Very often fast food, snacks and finger foods, sweet baked goods, processed meat products (e.g., sausage), red meat, full-fat dairy products, sweetened beverages, few freshly cooked meals, hardly any fruits and vegetables.	8 (1.9)	3 (2.1)	5 (1.8)	
Party preferences in general election (%)	Other party	2 (0.6)	1 (0.8)	1 (0.4)	0.829
	Bier Die Bierpartei	7 (1.9)	2 (1.6)	5 (2.1)	
	FPÖ Freiheitliche Partei Österreichs	15 (4.2)	7 (5.7)	8 (3.4)	
	GAZA Stimme gegen den Völkermord	3 (0.8)	2 (1.6)	1 (0.4)	
	Grüne Die Grüne Alternative	106 (29.4)	32 (26.2)	74 (31.1)	
	KEINE Keine von denen	5 (1.4)	1 (0.8)	4 (1.7)	
	KPÖ Kommunistische Partei Österreichs	27 (7.5)	8 (6.6)	19 (8.0)	
	LMP Liste Madeleine Petrovic	3 (0.8)	1 (0.8)	2 (0.8)	
	NEOS Die Reformkraft für dein neues Österreich	63 (17.5)	24 (19.7)	39 (16.4)	

Variables	Level	Overall	Male	Female	p
	ÖVP Die Volkspartei	24 (6.7)	10 (8.2)	14 (5.9)	
	SPÖ Sozialdemokratische Partei Österreichs	88 (24.4)	29 (23.8)	59 (24.8)	
	Would not vote at all	9 (2.5)	4 (3.3)	5 (2.1)	
	Would cast an invalid vote	8 (2.2)	1 (0.8)	7 (2.9)	
Party preferences grouped (%)	Left-wing	238 (66.1)	75 (61.5)	163 (68.5)	0.286
	Non-voter/Invalid	17 (4.7)	5 (4.1)	12 (5.0)	
	Right-wing	105 (29.2)	42 (34.4)	63 (26.5)	
Age (%)	15 to 19 years	5 (1.2)	1 (0.7)	4 (1.4)	0.060
	20 to 24 years	127 (29.6)	37 (25.9)	90 (31.5)	
	25 to 29 years	87 (20.3)	37 (25.9)	50 (17.5)	
	30 to 34 years	27 (6.3)	8 (5.6)	19 (6.6)	
	35 to 39 years	6 (1.4)	3 (2.1)	3 (1.0)	
	40 to 44 years	20 (4.7)	4 (2.8)	16 (5.6)	
	45 to 49 years	15 (3.5)	4 (2.8)	11 (3.8)	
	50 to 54 years	57 (13.3)	15 (10.5)	42 (14.7)	
	55 to 59 years	43 (10.0)	17 (11.9)	26 (9.1)	
	60 to 64 years	15 (3.5)	10 (7.0)	5 (1.7)	
	65	27 (6.3)	7 (4.9)	20 (7.0)	
Age grouped (%)	15–25	132 (30.8)	38 (26.6)	94 (32.9)	0.394
	25–49	155 (36.1)	56 (39.2)	99 (34.6)	
	Older than 50	142 (33.1)	49 (34.3)	93 (32.5)	
Education (%)	AHS with Matura (general secondary school with diploma)	108 (25.2)	40 (28.0)	68 (23.8)	0.003
	Other school	5 (1.2)	0 (0.0)	5 (1.7)	
	Bachelor’s	87 (20.3)	26 (18.2)	61 (21.3)	
	Professional Maturity Exam/Vocational Matura (evening school)	7 (1.6)	3 (2.1)	4 (1.4)	

Variables	Level	Overall	Male	Female	p
	BHS with Matura (e.g., HTL, HAK, HBLA, etc.)	47 (11.0)	23 (16.1)	24 (8.4)	
	BMS (vocational school, e.g., HASCH)	15 (3.5)	3 (2.1)	12 (4.2)	
	Doctorate / PhD	16 (3.7)	11 (7.7)	5 (1.7)	
	University-related educational institution or college	31 (7.2)	3 (2.1)	28 (9.8)	
	Apprenticeship, vocational school	21 (4.9)	7 (4.9)	14 (4.9)	
	Magister / Master's / Diplom-Ingenieur / University of Applied Sciences / Medical Studies	82 (19.1)	25 (17.5)	57 (19.9)	
	Polytechnic	1 (0.2)	0 (0.0)	1 (0.3)	
	University entrance examination	6 (1.4)	2 (1.4)	4 (1.4)	
	Primary school or less, lower secondary school, or lower AHS	3 (0.7)	0 (0.0)	3 (1.0)	
Education grouped (%)	Matura (school leaving exam)	193 (45.0)	69 (48.3)	124 (43.4)	0.267
	Above Matura	103 (24.0)	37 (25.9)	66 (23.1)	
	Below Matura	133 (31.0)	37 (25.9)	96 (33.6)	
Income per month (%)	I do not have my own income	41 (10.4)	20 (15.4)	21 (7.9)	<0.001
	Less than 250€	8 (2.0)	3 (2.3)	5 (1.9)	
	250-500€	38 (9.6)	7 (5.4)	31 (11.7)	
	500-1000€	50 (12.6)	21 (16.2)	29 (10.9)	
	1000-1500€	37 (9.3)	11 (8.5)	26 (9.8)	
	1500-2000€	45 (11.4)	7 (5.4)	38 (14.3)	
	2000-2500€	37 (9.3)	8 (6.2)	29 (10.9)	
	2500-3000€	41 (10.4)	10 (7.7)	31 (11.7)	

Variables	Level	Overall	Male	Female	p
	3000-3500€	28 (7.1)	3 (2.3)	25 (9.4)	
	3500-4000€	29 (7.3)	12 (9.2)	17 (6.4)	
	4000€ or more	42 (10.6)	28 (21.5)	14 (5.3)	
Income grouped (%)	Less than 1500 Euro	174 (43.9)	62 (47.7)	112 (42.1)	0.001
	1500–2999	123 (31.1)	25 (19.2)	98 (36.8)	
	More than 3000 Euro	99 (25.0)	43 (33.1)	56 (21.1)	
Have heard about anthroposophic medicine (%)	Yes, I can also explain what anthroposophic medicine means	63 (14.8)	21 (15.0)	42 (14.7)	0.982
	Yes, I am familiar with the term anthroposophic medicine, but I cannot explain it (or not sufficiently)	164 (38.5)	53 (37.9)	111 (38.8)	
	No, I have never heard of it	199 (46.7)	66 (47.1)	133 (46.5)	
Believe in anthroposophic medicine as a science (%)	Do not agree at all	103 (28.9)	49 (41.2)	54 (22.8)	0.002
	Agree less	105 (29.5)	33 (27.7)	72 (30.4)	
	Partially agree	114 (32.0)	26 (21.8)	88 (37.1)	
	Agree	34 (9.6)	11 (9.2)	23 (9.7)	
Believe in anthroposophic medicine as a science regrouped (%)	FALSE	208 (58.4)	82 (68.9)	126 (53.2)	0.006
	TRUE	148 (41.6)	37 (31.1)	111 (46.8)	
Believe that doctors trained in anthroposophy are more competent than other doctors (%)	Do not agree at all	137 (38.0)	62 (52.5)	75 (30.9)	0.001
	Agree less	87 (24.1)	25 (21.2)	62 (25.5)	
	Partially agree	97 (26.9)	20 (16.9)	77 (31.7)	
	Agree	40 (11.1)	11 (9.3)	29 (11.9)	
Alternative medicine sought (%)	Yes	95 (22.3)	20 (14.2)	75 (26.3)	0.007
	No	331 (77.7)	121 (85.8)	210 (73.7)	
Conventional medicine sought (%)	Yes	322 (76.5)	102 (72.9)	220 (78.3)	0.264
	No	99 (23.5)	38 (27.1)	61 (21.7)	

Variables	Level	Overall	Male	Female	p
Frequency of CAM-treatments (%)	Never	291 (68.0)	116 (81.1)	175 (61.4)	0.001
	Tried once	36 (8.4)	6 (4.2)	30 (10.5)	
	Rarely (once a year or less)	50 (11.7)	9 (6.3)	41 (14.4)	
	Occasionally (several times a year)	41 (9.6)	8 (5.6)	33 (11.6)	
	Regularly (monthly or more frequently)	10 (2.3)	4 (2.8)	6 (2.1)	
Frequency of CAM-treatment regrouped (%)	Occasionally/regularly	51 (11.9)	12 (8.4)	39 (13.6)	<0.001
	Only once/rarely	86 (20.0)	15 (10.5)	71 (24.8)	
	Never	292 (68.1)	116 (81.1)	176 (61.5)	
Preventive care (%)	Yes, I have taken preventive check-ups in the past and plan to continue doing so in the future	342 (79.9)	91 (64.1)	251 (87.8)	<0.001
	Yes, I have taken preventive check-ups in the past, but I do NOT plan to do so in the future	6 (1.4)	2 (1.4)	4 (1.4)	
	Yes, I plan to undergo preventive check-ups in the future, although I have not done so in the past	66 (15.4)	38 (26.8)	28 (9.8)	
	No, I have neither taken preventive check-ups in the past nor do I plan to in the future	14 (3.3)	11 (7.7)	3 (1.0)	
Importance of vaccination (%)	Do not agree at all	11 (2.6)	2 (1.4)	9 (3.2)	0.158
	Partially agree	82 (19.4)	20 (14.2)	62 (22.0)	
	Agree less	14 (3.3)	5 (3.5)	9 (3.2)	
	Agree	316 (74.7)	114 (80.9)	202 (71.6)	
Preventive care regrouped (%)	FALSE	87 (20.3)	52 (36.4)	35 (12.2)	<0.001

Variables	Level	Overall	Male	Female	p
	TRUE	342 (79.7)	91 (63.6)	251 (87.8)	
Importance of vaccination regrouped (%)	FALSE	113 (26.3)	29 (20.3)	84 (29.4)	0.058
	TRUE	316 (73.7)	114 (79.7)	202 (70.6)	
Knowledge of EBM(%)	Yes, I can also explain what EBM means	207 (49.6)	81 (58.3)	126 (45.3)	0.023
	Yes, I am familiar with the term EBM, but I cannot explain it (or not sufficiently)	116 (27.8)	36 (25.9)	80 (28.8)	
	No, I have never heard of it	94 (22.5)	22 (15.8)	72 (25.9)	
Study evidence is most important in therapy choice (%)	Do not agree at all	5 (1.2)	2 (1.4)	3 (1.1)	0.003
	Agree less	25 (5.9)	2 (1.4)	23 (8.1)	
	Partially agree	97 (22.9)	23 (16.5)	74 (26.0)	
	Agree	297 (70.0)	112 (80.6)	185 (64.9)	
Study evidence is most important in therapy choice - regrouped (%)	Do not agree/agree very little	30 (7.0)	4 (2.8)	26 (9.1)	0.027
	Agree/partially agree	399 (93.0)	139 (97.2)	260 (90.9)	
Age and eminence are most important in therapy choice (%)	Do not agree at all	53 (12.8)	25 (18.5)	28 (10.0)	0.052
	Agree less	118 (28.4)	41 (30.4)	77 (27.5)	
	Partially agree	185 (44.6)	54 (40.0)	131 (46.8)	
	Agree	59 (14.2)	15 (11.1)	44 (15.7)	
Age and eminence are most important in therapy choice - regrouped (%)	Do not agree/agree very little	171 (39.9)	66 (46.2)	105 (36.7)	0.075
	Agree/partially agree	258 (60.1)	77 (53.8)	181 (63.3)	
Finished our survey (%)	0	8 (1.9)	4 (2.8)	4 (1.4)	0.528
	1	421 (98.1)	139 (97.2)	282 (98.6)	
Missing percent (mean (SD))		1.50 (3.25)	1.90 (4.93)	1.30 (1.92)	0.071
Completion speed (mean (SD))		1.11 (0.41)	1.20 (0.41)	1.07 (0.40)	0.002
Country (%)	Other country	7 (1.6)	4 (2.8)	3 (1.0)	0.192
	Germany	13 (3.0)	7 (4.9)	6 (2.1)	

Variables	Level	Overall	Male	Female	p
	Austria	407 (94.9)	131 (91.6)	276 (96.5)	
	Switzerland	2 (0.5)	1 (0.7)	1 (0.3)	
