# Supplementary material for: Authoritarian attitudes and the perceived scientific legitimacy of anthroposophic medicine: A survey of attitudes on complementary and alternative medicine in Austria
Source: PLoS One. 2026 Jun 17;21(6):e0348672. doi: 10.1371/journal.pone.0348672 (PMC13274894; doi:10.1371/journal.pone.0348672)
Supplement: S2 Table — All measures stratified by leaning towards conventional vs. CAM. (PDF) [file pone.0348672.s002.pdf]

Supplement 2: All measures stratified by leaning towards conventional vs. complementary and alternative medicine

Variables	Level	Overall	Favours conventional	Favours alternative	p
n		429	282	147	
Satisfaction with conventional medicine (mean (SD))		2.00 (0.05)	2.00 (0.06)	2.00 (0.00)	0.469
Satisfaction with alternative medicine (mean (SD))		1.50 (0.50)	1.35 (0.48)	1.78 (0.42)	<0.001
Satisfaction with complementary medicine (mean (SD))		1.61 (0.49)	1.46 (0.50)	1.89 (0.31)	<0.001
Overall life satisfaction (mean (SD))		3.71 (0.71)	3.72 (0.70)	3.68 (0.72)	0.644
Positive outlook for the future (mean (SD))		3.39 (0.88)	3.34 (0.87)	3.49 (0.91)	0.110
Regular worries in life (mean (SD))		3.10 (1.03)	3.13 (1.00)	3.03 (1.09)	0.316
Spiritual beliefs help with difficulties (mean (SD))		2.68 (1.27)	2.46 (1.24)	3.11 (1.23)	<0.001
Mean (±SD) response score: Average rating for a migraine case scenario on a 0–100 scale (0 = conventional medicine; 100 = CAM), with variability indicated by the standard deviation		62.23 (23.73)	74.76 (16.55)	38.19 (15.44)	<0.001
Perceived control over health (%)	1	0 (0)	0 (0)	0 (0)	0.293
	2	27 (6.4)	20 (7.3)	7 (4.8)	
	3	65 (15.5)	46 (16.8)	19 (13.0)	
	4	255 (60.7)	166 (60.6)	89 (61.0)	
	5	73 (17.4)	42 (15.3)	31 (21.2)	
Illness perceived as a matter of luck (rather than misfortune) (%)	1	66 (15.8)	31 (11.3)	35 (24.3)	0.006
	2	164 (39.1)	107 (38.9)	57 (39.6)	
	3	85 (20.3)	61 (22.2)	24 (16.7)	
	4	93 (22.2)	67 (24.4)	26 (18.1)	
	5	11 (2.6)	9 (3.3)	2 (1.4)	
Tendency to reassure oneself and accept the situation rather than acting (%)	1	13 (3.1)	8 (2.9)	5 (3.5)	0.678

Variables	Level	Overall	Favours conventional	Favours alternative	p
	2	64 (15.3)	40 (14.7)	24 (16.7)	
	3	91 (21.8)	55 (20.1)	36 (25.0)	
	4	189 (45.3)	130 (47.6)	59 (41.0)	
	5	60 (14.4)	40 (14.7)	20 (13.9)	
Active approach to resolving the cause of distress instead of just self-soothing (%)	1	0 (0)	0 (0)	0 (0)	0.155
	2	24 (5.7)	19 (6.9)	5 (3.4)	
	3	45 (10.7)	32 (11.6)	13 (9.0)	
	4	231 (54.9)	154 (55.8)	77 (53.1)	
	5	121 (28.7)	71 (25.7)	50 (34.5)	
Self-blame for illness (%)	1	61 (14.5)	41 (14.9)	20 (13.8)	0.064
	2	186 (44.2)	131 (47.5)	55 (37.9)	
	3	100 (23.8)	65 (23.6)	35 (24.1)	
	4	70 (16.6)	38 (13.8)	32 (22.1)	
	5	4 (1.0)	1 (0.4)	3 (2.1)	
Every problem has a solution (%)	1	13 (3.1)	8 (2.9)	5 (3.4)	0.034
	2	43 (10.2)	36 (13.1)	7 (4.8)	
	3	55 (13.1)	31 (11.3)	24 (16.4)	
	4	239 (56.8)	159 (57.8)	80 (54.8)	
	5	71 (16.9)	41 (14.9)	30 (20.5)	
Ability to cope well with events (%)	1	4 (1.0)	3 (1.1)	1 (0.7)	0.368
	2	27 (6.4)	22 (8.0)	5 (3.4)	
	3	68 (16.2)	44 (16.0)	24 (16.4)	
	4	242 (57.5)	158 (57.5)	84 (57.5)	
	5	80 (19.0)	48 (17.5)	32 (21.9)	
Adjusting daily life for health (%)	1	2 (0.5)	1 (0.4)	1 (0.7)	0.024
	2	26 (6.2)	23 (8.4)	3 (2.1)	
	3	40 (9.6)	26 (9.5)	14 (9.7)	
	4	214 (51.3)	146 (53.5)	68 (47.2)	
	5	135 (32.4)	77 (28.2)	58 (40.3)	
Refraining from indulgences for health (%)	1	3 (0.7)	3 (1.1)	0 (0.0)	0.135

Variables	Level	Overall	Favours conventional	Favours alternative	p
	2	42 (10.1)	34 (12.5)	8 (5.6)	
	3	40 (9.6)	26 (9.6)	14 (9.7)	
	4	204 (49.0)	127 (46.7)	77 (53.5)	
	5	127 (30.5)	82 (30.1)	45 (31.2)	
Endorsing the notion that sharing similar values is beneficial (%)	1	55 (14.3)	35 (14.2)	20 (14.6)	0.365
	2	66 (17.2)	41 (16.6)	25 (18.2)	
	3	76 (19.8)	53 (21.5)	23 (16.8)	
	4	118 (30.7)	80 (32.4)	38 (27.7)	
	5	69 (18.0)	38 (15.4)	31 (22.6)	
Belief that experts who cannot give clear answers likely lack knowledge (%)	1	151 (36.8)	117 (43.7)	34 (23.9)	<0.001
	2	130 (31.7)	84 (31.3)	46 (32.4)	
	3	62 (15.1)	36 (13.4)	26 (18.3)	
	4	54 (13.2)	24 (9.0)	30 (21.1)	
	5	13 (3.2)	7 (2.6)	6 (4.2)	
Preference for strong leaders (%)	1	76 (18.6)	52 (19.4)	24 (17.0)	0.736
	2	103 (25.2)	71 (26.5)	32 (22.7)	
	3	87 (21.3)	55 (20.5)	32 (22.7)	
	4	117 (28.6)	72 (26.9)	45 (31.9)	
	5	26 (6.4)	18 (6.7)	8 (5.7)	
Not questioning tried-and-tested behaviors (%)	1	149 (36.1)	110 (41.0)	39 (26.9)	0.031
	2	145 (35.1)	91 (34.0)	54 (37.2)	
	3	58 (14.0)	34 (12.7)	24 (16.6)	
	4	54 (13.1)	28 (10.4)	26 (17.9)	
	5	7 (1.7)	5 (1.9)	2 (1.4)	
Number of comorbidities (mean (SD)		1.94 (1.45)	2.06 (1.52)	1.72 (1.29)	0.022
Comorbidities (%)	FALSE	103 (24.6)	64 (23.3)	39 (27.1)	0.459
	TRUE	316 (75.4)	211 (76.7)	105 (72.9)	
Recent infections (%)	Checked	194 (45.2)	131 (46.5)	63 (42.9)	0.543
	Not checked	235 (54.8)	151 (53.5)	84 (57.1)	

Variables	Level	Overall	Favours conventional	Favours alternative	p
Comorbidity: Cancer (%)	Checked	9 (2.1)	5 (1.8)	4 (2.7)	0.768
	Not checked	420 (97.9)	277 (98.2)	143 (97.3)	
Blood/immune system conditions (%)	Checked	16 (3.7)	11 (3.9)	5 (3.4)	1.000
	Not checked	413 (96.3)	271 (96.1)	142 (96.6)	
Nutritional/metabolic diseases (%)	Checked	33 (7.7)	26 (9.2)	7 (4.8)	0.146
	Not checked	396 (92.3)	256 (90.8)	140 (95.2)	
Nervous system conditions (%)	Checked	11 (2.6)	6 (2.1)	5 (3.4)	0.638
	Not checked	418 (97.4)	276 (97.9)	142 (96.6)	
Psychological conditions (%)	Checked	74 (17.2)	57 (20.2)	17 (11.6)	0.034
	Not checked	355 (82.8)	225 (79.8)	130 (88.4)	
Eye conditions (%)	Checked	26 (6.1)	18 (6.4)	8 (5.4)	0.862
	Not checked	403 (93.9)	264 (93.6)	139 (94.6)	
Ear conditions (%)	Checked	21 (4.9)	15 (5.3)	6 (4.1)	0.743
	Not checked	408 (95.1)	267 (94.7)	141 (95.9)	
Cardiovascular conditions (%)	Checked	20 (4.7)	17 (6.0)	3 (2.0)	0.106
	Not checked	409 (95.3)	265 (94.0)	144 (98.0)	
Respiratory conditions (%)	Checked	46 (10.7)	34 (12.1)	12 (8.2)	0.283
	Not checked	383 (89.3)	248 (87.9)	135 (91.8)	
Digestive system conditions (%)	Checked	53 (12.4)	40 (14.2)	13 (8.8)	0.150
	Not checked	376 (87.6)	242 (85.8)	134 (91.2)	
Skin conditions (%)	Checked	56 (13.1)	41 (14.5)	15 (10.2)	0.265
	Not checked	373 (86.9)	241 (85.5)	132 (89.8)	
Musculoskeletal conditions (%)	Checked	55 (12.8)	32 (11.3)	23 (15.6)	0.266
	Not checked	374 (87.2)	250 (88.7)	124 (84.4)	
Urogenital conditions (%)	Checked	36 (8.4)	24 (8.5)	12 (8.2)	1.000
	Not checked	393 (91.6)	258 (91.5)	135 (91.8)	
Congenital malformations (%)	Checked	6 (1.4)	5 (1.8)	1 (0.7)	0.630
	Not checked	423 (98.6)	277 (98.2)	146 (99.3)	
Injuries (%)	Checked	79 (18.4)	60 (21.3)	19 (12.9)	0.047
	Not checked	350 (81.6)	222 (78.7)	128 (87.1)	
No comorbidities (%)	Checked	74 (17.2)	44 (15.6)	30 (20.4)	0.265

Variables	Level	Overall	Favours conventional	Favours alternative	p
	Not checked	355 (82.8)	238 (84.4)	117 (79.6)	
Prefer not to answer – comorbidities (%)	Checked	10 (2.3)	7 (2.5)	3 (2.0)	1.000
	Not checked	419 (97.7)	275 (97.5)	144 (98.0)	
Other diseases (%)	Checked	17 (4.0)	9 (3.2)	8 (5.4)	0.382
	Not checked	412 (96.0)	273 (96.8)	139 (94.6)	
Taken seriously by healthcare professionals (%)	I was almost always taken seriously	139 (32.8)	102 (36.4)	37 (25.7)	0.259
	I was mostly taken seriously	146 (34.4)	93 (33.2)	53 (36.8)	
	Half and half	124 (29.2)	76 (27.1)	48 (33.3)	
	I was almost never taken seriously	2 (0.5)	1 (0.4)	1 (0.7)	
	I was mostly NOT taken seriously	13 (3.1)	8 (2.9)	5 (3.5)	
Taken seriously by healthcare professionals - different grouping (%)	I was almost always taken seriously	139 (32.4)	102 (36.2)	37 (25.2)	0.058
	I was mostly taken seriously	146 (34.0)	93 (33.0)	53 (36.1)	
	Some to little	144 (33.6)	87 (30.9)	57 (38.8)	
Money spent on evidence-based medicine (%)	0€	123 (29.4)	92 (33.3)	31 (21.8)	0.005
	1-100€	36 (8.6)	29 (10.5)	7 (4.9)	
	100-250€	85 (20.3)	58 (21.0)	27 (19.0)	
	250-500€	78 (18.7)	41 (14.9)	37 (26.1)	
	500-750€	47 (11.2)	28 (10.1)	19 (13.4)	
	750-1000€	23 (5.5)	11 (4.0)	12 (8.5)	
	1000€ or more	26 (6.2)	17 (6.2)	9 (6.3)	
Money spent on eminence-based medicine (%)	0€	276 (65.6)	215 (77.6)	61 (42.4)	<0.001
	1-100€	48 (11.4)	28 (10.1)	20 (13.9)	
	100-250€	38 (9.0)	17 (6.1)	21 (14.6)	
	250-500€	27 (6.4)	6 (2.2)	21 (14.6)	
	500-750€	15 (3.6)	5 (1.8)	10 (6.9)	

Variables	Level	Overall	Favours conventional	Favours alternative	p
	750-1000€	8 (1.9)	2 (0.7)	6 (4.2)	
	1000€ or more	9 (2.1)	4 (1.4)	5 (3.5)	
Location (%)	Countryside	123 (30.5)	80 (30.2)	43 (31.2)	0.931
	City	280 (69.5)	185 (69.8)	95 (68.8)	
Sports (%)	No sporting activity	36 (8.6)	21 (7.6)	15 (10.6)	0.253
	Less than 1 hour per week	57 (13.6)	36 (13.0)	21 (14.8)	
	Regularly, 1–2 hours per week	126 (30.1)	93 (33.6)	33 (23.2)	
	Regularly, 2–4 hours per week	122 (29.1)	79 (28.5)	43 (30.3)	
	Regularly, more than 4 hours per week	78 (18.6)	48 (17.3)	30 (21.1)	
Nutrition (%)	Predominantly plant-based and freshly cooked meals, legumes (e.g., lentils, beans), lots of fruits and vegetables, whole grain products, fish, healthy fats (e.g., nuts, avocado), hardly any snacks or finger foods, hardly any sweet baked goods.	91 (21.4)	60 (21.5)	31 (21.2)	0.909
	Regularly plant-based and freshly cooked meals, legumes (e.g., lentils, beans), often fruits and vegetables, whole grain products, fish, healthy fats (e.g., nuts, avocado), occasionally snacks and finger foods, occasionally sweet baked goods.	279 (65.6)	181 (64.9)	98 (67.1)	

Variables	Level	Overall	Favours conventional	Favours alternative	p
	Often fast food, snacks and finger foods, sweet baked goods, processed meat products (e.g., sausage), red meat, full-fat dairy products, sweetened beverages, sometimes freshly cooked meals, occasionally fruits and vegetables.	47 (11.1)	33 (11.8)	14 (9.6)	
	Very often fast food, snacks and finger foods, sweet baked goods, processed meat products (e.g., sausage), red meat, full-fat dairy products, sweetened beverages, few freshly cooked meals, hardly any fruits and vegetables.	8 (1.9)	5 (1.8)	3 (2.1)	
Party preferences in general election (%)	Other party	2 (0.6)	1 (0.4)	1 (0.9)	0.089
	Bier Die Bierpartei	7 (1.9)	5 (2.0)	2 (1.8)	
	FPÖ Freiheitliche Partei Österreichs	15 (4.2)	9 (3.7)	6 (5.3)	
	GAZA Stimme gegen den Völkermord	3 (0.8)	2 (0.8)	1 (0.9)	
	Grüne Die Grüne Alternative	106 (29.4)	80 (32.5)	26 (22.8)	
	KEINE Keine von denen	5 (1.4)	2 (0.8)	3 (2.6)	
	KPÖ Kommunistische Partei Österreichs	27 (7.5)	19 (7.7)	8 (7.0)	
	LMP Liste Madeleine Petrovic	3 (0.8)	0 (0.0)	3 (2.6)	

Variables	Level	Overall	Favours conventional	Favours alternative	p
	NEOS Die Reformkraft für dein neues Österreich	63 (17.5)	39 (15.9)	24 (21.1)	
	ÖVP Die Volkspartei	24 (6.7)	17 (6.9)	7 (6.1)	
	SPÖ Sozialdemokratische Partei Österreichs	88 (24.4)	64 (26.0)	24 (21.1)	
	would not vote at all	9 (2.5)	3 (1.2)	6 (5.3)	
	would cast an invalid vote	8 (2.2)	5 (2.0)	3 (2.6)	
Party preferences grouped (%)	Left-wing	238 (66.1)	173 (70.3)	65 (57.0)	0.022
	Non-voter/Invalid	17 (4.7)	8 (3.3)	9 (7.9)	
	Right-wing	105 (29.2)	65 (26.4)	40 (35.1)	
Age (%)	15 to 19 years	5 (1.2)	5 (1.8)	0 (0.0)	<0.001
	20 to 24 years	127 (29.6)	99 (35.1)	28 (19.0)	
	25 to 29 years	87 (20.3)	69 (24.5)	18 (12.2)	
	30 to 34 years	27 (6.3)	16 (5.7)	11 (7.5)	
	35 to 39 years	6 (1.4)	3 (1.1)	3 (2.0)	
	40 to 44 years	20 (4.7)	9 (3.2)	11 (7.5)	
	45 to 49 years	15 (3.5)	6 (2.1)	9 (6.1)	
	50 to 54 years	57 (13.3)	29 (10.3)	28 (19.0)	
	55 to 59 years	43 (10.0)	22 (7.8)	21 (14.3)	
	60 to 64 years	15 (3.5)	7 (2.5)	8 (5.4)	
	65 and older	27 (6.3)	17 (6.0)	10 (6.8)	
Age grouped (%)	15–25	132 (30.8)	104 (36.9)	28 (19.0)	<0.001
	25–49	155 (36.1)	103 (36.5)	52 (35.4)	
	Older than 50	142 (33.1)	75 (26.6)	67 (45.6)	
Education (%)	AHS with Matura (general secondary school with diploma)	108 (25.2)	87 (30.9)	21 (14.3)	0.002
	Other school	5 (1.2)	3 (1.1)	2 (1.4)	
	Bachelor’s	87 (20.3)	59 (20.9)	28 (19.0)	

Variables	Level	Overall	Favours conventional	Favours alternative	p
	Professional Maturity Exam/Vocational Matura (evening school)	7 (1.6)	4 (1.4)	3 (2.0)	
	BHS with Matura (e.g., HTL, HAK, HBLA, etc.)	47 (11.0)	24 (8.5)	23 (15.6)	
	BMS (vocational school, e.g., HASCH)	15 (3.5)	8 (2.8)	7 (4.8)	
	Doctorate / PhD	16 (3.7)	12 (4.3)	4 (2.7)	
	University-related educational institution or college	31 (7.2)	11 (3.9)	20 (13.6)	
	Apprenticeship, vocational school	21 (4.9)	14 (5.0)	7 (4.8)	
	Magister / Master's / Diplom-Ingenieur / University of Applied Sciences / Medical Studies	82 (19.1)	54 (19.1)	28 (19.0)	
	Polytechnic	1 (0.2)	0 (0.0)	1 (0.7)	
	University entrance examination	6 (1.4)	4 (1.4)	2 (1.4)	
	Primary school or below, below secondary school, or below AHS	3 (0.7)	2 (0.7)	1 (0.7)	
Education grouped (%)	Matura (school leaving exam)	193 (45.0)	126 (44.7)	67 (45.6)	0.711
	Above Matura	103 (24.0)	71 (25.2)	32 (21.8)	
	Below Matura	133 (31.0)	85 (30.1)	48 (32.7)	
Income per month (%)	I do not have my own income	41 (10.4)	36 (13.7)	5 (3.7)	<0.001
	Less than 250€	8 (2.0)	5 (1.9)	3 (2.2)	
	250-500€	38 (9.6)	33 (12.6)	5 (3.7)	
	500-1000€	50 (12.6)	39 (14.9)	11 (8.2)	
	1000-1500€	37 (9.3)	24 (9.2)	13 (9.7)	

Variables	Level	Overall	Favours conventional	Favours alternative	p
	1500-2000€	45 (11.4)	25 (9.5)	20 (14.9)	
	2000-2500€	37 (9.3)	20 (7.6)	17 (12.7)	
	2500-3000€	41 (10.4)	24 (9.2)	17 (12.7)	
	3000-3500€	28 (7.1)	16 (6.1)	12 (9.0)	
	3500-4000€	29 (7.3)	13 (5.0)	16 (11.9)	
	4000€ or more	42 (10.6)	27 (10.3)	15 (11.2)	
Income grouped (%)	Less than 1500 Euro	174 (43.9)	137 (52.3)	37 (27.6)	<0.001
	1500–2999	123 (31.1)	69 (26.3)	54 (40.3)	
	More than 3000 Euro	99 (25.0)	56 (21.4)	43 (32.1)	
Gender (%)	Male	143 (33.3)	106 (37.6)	37 (25.2)	0.013
	Female	286 (66.7)	176 (62.4)	110 (74.8)	
Have heard about anthroposophic medicine (%)	Yes, I can also explain what anthroposophic medicine means	63 (14.8)	41 (14.7)	22 (15.0)	0.602
	Yes, I am familiar with the term anthroposophic medicine, but I cannot explain it (or not sufficiently)	164 (38.5)	103 (36.9)	61 (41.5)	
	No, I have never heard of it	199 (46.7)	135 (48.4)	64 (43.5)	
Believe in anthroposophic medicine as a science (%)	Do not agree at all	103 (28.9)	91 (38.2)	12 (10.2)	<0.001
	Agree less	105 (29.5)	75 (31.5)	30 (25.4)	
	Partially agree	114 (32.0)	62 (26.1)	52 (44.1)	
	Agree	34 (9.6)	10 (4.2)	24 (20.3)	
Believe in antrhoposophic medicine as a science regrouped (%)	FALSE	208 (58.4)	166 (69.7)	42 (35.6)	<0.001
	TRUE	148 (41.6)	72 (30.3)	76 (64.4)	
Believe that doctors trained in anthroposophy are more competent than other doctors (%)	Do not agree at all	137 (38.0)	117 (49.2)	20 (16.3)	<0.001

Variables	Level	Overall	Favours conventional	Favours alternative	p
	Agree less	87 (24.1)	62 (26.1)	25 (20.3)	
	Partially agree	97 (26.9)	46 (19.3)	51 (41.5)	
	Agree	40 (11.1)	13 (5.5)	27 (22.0)	
Alternative medicine sought (%)	Yes	95 (22.3)	38 (13.6)	57 (38.8)	<0.001
	No	331 (77.7)	241 (86.4)	90 (61.2)	
Conventional medicine sought (%)	Yes	322 (76.5)	212 (76.5)	110 (76.4)	1.000
	No	99 (23.5)	65 (23.5)	34 (23.6)	
Frequency of CAM-treatments (%)	Regularly (monthly or more frequently)	10 (2.3)	3 (1.1)	7 (4.8)	<0.001
	Occasionally (several times a year)	41 (9.6)	19 (6.7)	22 (15.1)	
	Rarely (once a year or less)	50 (11.7)	24 (8.5)	26 (17.8)	
	Tried once	36 (8.4)	25 (8.9)	11 (7.5)	
	Never	291 (68.0)	211 (74.8)	80 (54.8)	
Frequency of CAM-treatments regrouped (%)	Occasionally/regularly	51 (11.9)	22 (7.8)	29 (19.7)	<0.001
	Only once/rarely	86 (20.0)	49 (17.4)	37 (25.2)	
	Never	292 (68.1)	211 (74.8)	81 (55.1)	
Preventive care (%)	Yes, I have taken preventive check-ups in the past and plan to continue doing so in the future	342 (79.9)	214 (76.2)	128 (87.1)	0.004
	Yes, I have taken preventive check-ups in the past, but I do NOT plan to do so in the future	6 (1.4)	2 (0.7)	4 (2.7)	
	Yes, I plan to undergo preventive check-ups in the future, although I have not done so in the past	66 (15.4)	55 (19.6)	11 (7.5)	

Variables	Level	Overall	Favours conventional	Favours alternative	p
	No, I have neither taken preventive check-ups in the past nor do I plan to in the future	14 (3.3)	10 (3.6)	4 (2.7)	
Importance of vaccination (%)	Do not agree at all	11 (2.6)	1 (0.4)	10 (7.0)	<0.001
	Agree less	14 (3.3)	2 (0.7)	12 (8.4)	
	Partially agree	82 (19.4)	40 (14.3)	42 (29.4)	
	Agree	316 (74.7)	237 (84.6)	79 (55.2)	
Preventive care regrouped (%)	FALSE	87 (20.3)	68 (24.1)	19 (12.9)	0.009
	TRUE	342 (79.7)	214 (75.9)	128 (87.1)	
Importance of vaccination regrouped (%)	FALSE	113 (26.3)	45 (16.0)	68 (46.3)	<0.001
	TRUE	316 (73.7)	237 (84.0)	79 (53.7)	
Knowledge of EBM(%)	Yes, I can also explain what EBM means	207 (49.6)	165 (59.4)	42 (30.2)	<0.001
	Yes, I am familiar with the term EBM, but I cannot explain it (or not sufficiently)	116 (27.8)	68 (24.5)	48 (34.5)	
	No, I have never heard of it	94 (22.5)	45 (16.2)	49 (35.3)	
Study evidence is most important in therapy choice (%)	Do not agree at all	5 (1.2)	1 (0.4)	4 (2.8)	<0.001
	Agree less	25 (5.9)	9 (3.2)	16 (11.2)	
	Partially agree	97 (22.9)	44 (15.7)	53 (37.1)	
	Agree	297 (70.0)	227 (80.8)	70 (49.0)	
Study evidence is most important in therapy choice regrouped (%)	Do not agree/agree very little	30 (7.0)	10 (3.5)	20 (13.6)	<0.001
	Agree/partially agree	399 (93.0)	272 (96.5)	127 (86.4)	
Age and eminence are most important in therapy choice (%)	Do not agree at all	53 (12.8)	46 (17.0)	7 (4.9)	<0.001
	Agree less	118 (28.4)	95 (35.1)	23 (16.0)	
	Partially agree	185 (44.6)	101 (37.3)	84 (58.3)	
	Agree	59 (14.2)	29 (10.7)	30 (20.8)	

Variables	Level	Overall	Favours conventional	Favours alternative	p
Age and eminence are most important in therapy choice regrouped (%)	Do not agree/agree very little	171 (39.9)	141 (50.0)	30 (20.4)	<0.001
	Agree/partially agree	258 (60.1)	141 (50.0)	117 (79.6)	
Has interview finished (%)	0	8 (1.9)	6 (2.1)	2 (1.4)	0.856
	1	421 (98.1)	276 (97.9)	145 (98.6)	
Missing percent (mean (SD))		1.50 (3.25)	1.63 (3.89)	1.24 (1.33)	0.239
Completion Speed (mean (SD))		1.11 (0.41)	1.16 (0.43)	1.02 (0.35)	0.001
Country (%)	Other country	7 (1.6)	5 (1.8)	2 (1.4)	0.485
	Germany	13 (3.0)	11 (3.9)	2 (1.4)	
	Austria	407 (94.9)	265 (94.0)	142 (96.6)	
	Switzerland	2 (0.5)	1 (0.4)	1 (0.7)	
