# Supplementary material for: Authoritarian attitudes and the perceived scientific legitimacy of anthroposophic medicine: A survey of attitudes on complementary and alternative medicine in Austria
Source: PLoS One. 2026 Jun 17;21(6):e0348672. doi: 10.1371/journal.pone.0348672 (PMC13274894; doi:10.1371/journal.pone.0348672)
Supplement: S3 Table — Regression model of sociodemographic factors. (PDF) [file pone.0348672.s003.pdf]

Supplement 3: Regression model of sociodemographic factors

Model	Variable	Estimate	Std. Error	t value	p-value	2.5 %	97.5 %
Original	Intercept	76.522	4.4930	17.032	<0.001	67.716	85.328
	Age: 25–49	-5.274	3.417	-1.544	0.124	-11.971	1.423
	Age: over 50	-18.653	4.241	-4.398	<0.001	-26.966	-10.341
	Education: Above high school	-3.145	3.137	-1.002	0.317	-9.293	3.004
	Education: Below high school	2.162	3.252	0.665	0.507	-4.211	8.535
	Income: More than €3000	3.034	3.553	0.854	0.394	-3.929	9.996
	Income: Less than €1500	3.932	3.410	1.153	0.250	-2.751	10.615
	Gender: Female	-10.530	2.673	-3.940	<0.001	-15.768	-5.291
	Vote: Non-voter/Invalid	-5.259	6.479	-0.812	0.418	-17.958	7.441
	Vote: Right	0.892	4.263	0.209	0.834	-7.463	9.247
Poststratified	Intercept	72.984	4.613	15.821	<0.001	63.943	82.026
	Age: 25–49	-7.280	3.800	-1.916	0.056	-14.727	0.167
	Age: Over 50	-8.967	4.424	-2.027	0.043	-17.637	-0.297
	Education: Above high school	-2.091	2.948	-0.709	0.479	-7.869	3.687
	Education: Below high school	1.430	3.110	0.460	0.646	-4.665	7.525
	Income: More than €3000	-0.258	3.063	-0.084	0.933	-6.261	5.746

Model	Variable	Estimate	Std. Error	t value	p-value	2.5 %	97.5 %
	Income: Less than €1500	6.517	3.406	1.913	0.057	-0.158	13.192
	Gender: Female	-7.430	2.463	-3.017	0.003	-12.257	-2.602
	Vote: Non-voter/Invalid	-2.815	3.572	-0.788	0.431	-9.816	4.185
	Vote: Right	-2.288	2.658	-0.860	0.390	-7.498	2.921
