# Supplementary material for: Authoritarian attitudes and the perceived scientific legitimacy of anthroposophic medicine: A survey of attitudes on complementary and alternative medicine in Austria
Source: PLoS One. 2026 Jun 17;21(6):e0348672. doi: 10.1371/journal.pone.0348672 (PMC13274894; doi:10.1371/journal.pone.0348672)
Supplement: S4 Table — Regression model of psychological predictors. (PDF) [file pone.0348672.s004.pdf]

Supplement 4: Regression model of psychological predictors

Model	Variable	Estimate	Std. Error	t value	p-value	2.5 %	97.5 %
Original	Intercept	-24.971	10.665	-2.341	0.020	-45.874	-4.068
	Perceived QoL	1.041	0.656	1.585	0.114	-0.246	2.327
	Health control	2.490	0.662	3.759	<0.001	1.192	3.788
	Stress coping	-0.587	0.968	-0.607	0.544	-2.484	1.310
	Self-efficacy	0.211	0.958	0.220	0.826	-1.666	2.088
	Health priority	1.778	0.828	2.146	0.033	0.154	3.401
	Ambiguity tolerance	1.658	0.664	2.496	0.013	0.356	2.959
	Fascist tendency	1.716	0.705	2.434	0.015	0.334	3.098
Poststratified	Intercept	0.953	10.557	0.090	0.928	-19.738	21.644
	Perceived QoL	-0.413	0.670	-0.617	0.538	-1.726	0.899
	Health control	2.229	0.642	3.474	0.001	0.971	3.487
	Stress coping	-2.546	1.065	-2.391	0.017	-4.632	-0.459
	Self-efficacy	0.736	0.953	0.772	0.441	-1.132	2.603
	Health priority	2.058	0.813	2.529	0.012	0.463	3.652
	Ambiguity tolerance	2.449	0.763	3.208	0.001	0.953	3.945
	Fascist tendency	0.610	0.719	0.848	0.397	-0.800	2.020
