# Supplementary material for: Authoritarian attitudes and the perceived scientific legitimacy of anthroposophic medicine: A survey of attitudes on complementary and alternative medicine in Austria
Source: PLoS One. 2026 Jun 17;21(6):e0348672. doi: 10.1371/journal.pone.0348672 (PMC13274894; doi:10.1371/journal.pone.0348672)

Supplement 5: Differences of Pre- and Poststratified Data in Authoritarian Orientation

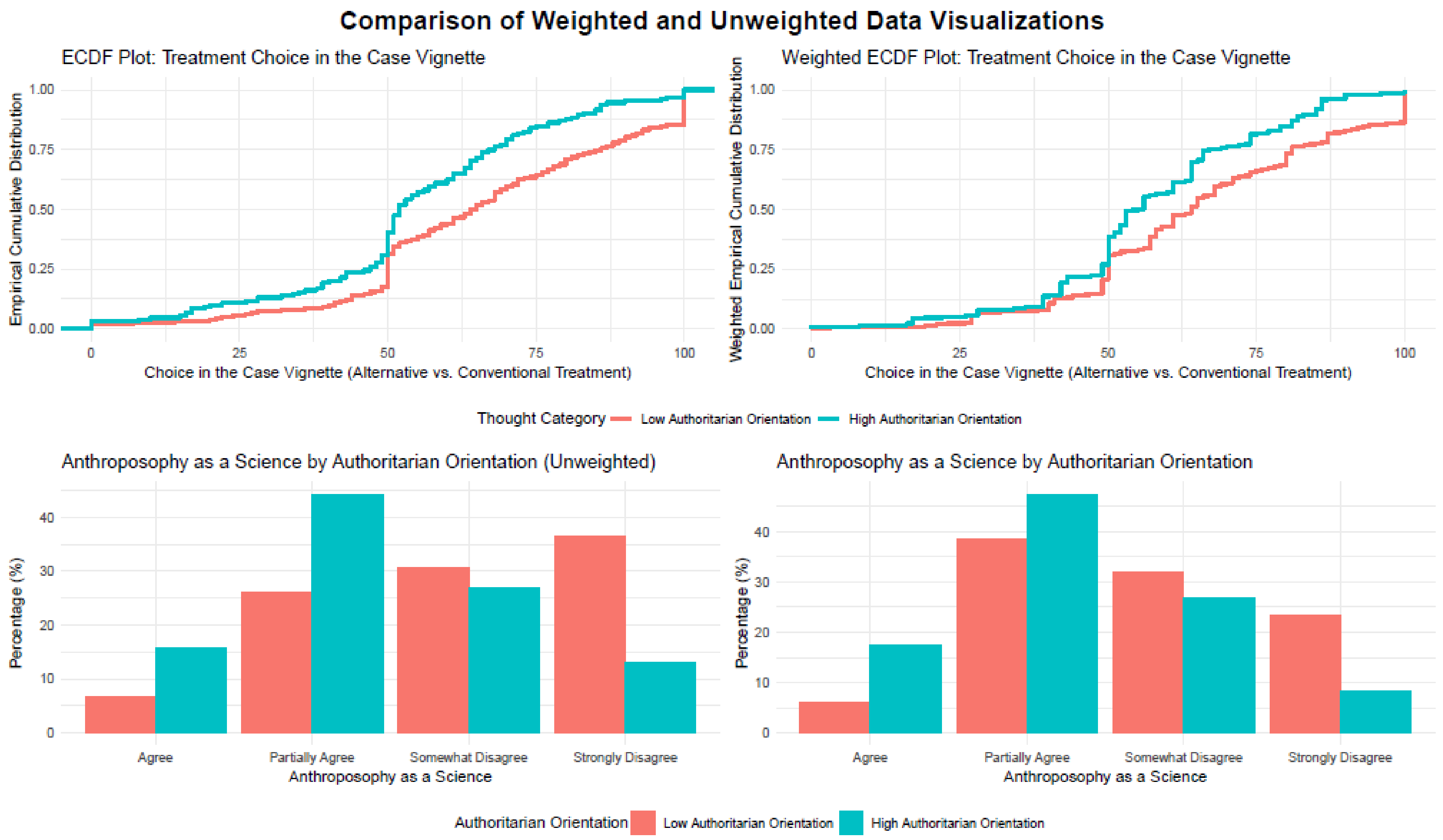

Supplement: S1 Fig — Differences of pre- and post-stratified data in authoritarian orientation. (PDF) [file pone.0348672.s005.pdf]
