# Supplementary material for: Authoritarian attitudes and the perceived scientific legitimacy of anthroposophic medicine: A survey of attitudes on complementary and alternative medicine in Austria
Source: PLoS One. 2026 Jun 17;21(6):e0348672. doi: 10.1371/journal.pone.0348672 (PMC13274894; doi:10.1371/journal.pone.0348672)
Supplement: S1 File — Survey questions. (PDF) [file pone.0348672.s006.pdf]

Supplement 6: Survey questions

Dear Participant,

On behalf of our diploma thesis project entitled **“Complementary Medicine: Esotericism & Evidence”**, we sincerely thank you for taking the time to participate in our survey.

Please note:

- Complete the questionnaire only once.
- There are no right or wrong answers.
- We kindly ask that you answer all questions as openly and honestly as possible; this will greatly support our research work.

Enjoy filling out the questionnaire!

All data provided will be handled confidentially and processed anonymously. No data are stored permanently. This study is conducted by students for research purposes only and does not serve any commercial interests or present any conflicts of interest.

Data Protection

Data Protection (Interactive Button)

1. **I hereby confirm that I have read and understood the data protection notice.**
   (Free-response field)

Preface to the Questionnaire

In this survey we aim to gather your opinions on conventional medicine (often referred to colloquially as “school medicine”) as well as alternative and complementary medicine. To help you understand the context, please review the brief definitions below:

Key Terms

- **Conventional Medicine:**
  Often called "school medicine," this form of medicine is characterized by its adherence to the latest scientific standards. It employs state-of-the-art technology, medications, and surgical procedures for diagnosis, treatment, and prevention of illnesses and injuries.
- **Alternative Medicine:**
  This term refers to treatment methods that are usually based on traditional, natural, or unconventional approaches. It includes practices such as acupuncture, homeopathy, herbal medicine, Traditional Chinese Medicine, among others, and is often used as an alternative to conventional treatments.
- **Complementary Medicine (e.g., Anthroposophic Medicine):**
  These are treatment methods used in addition to conventional medical treatments to support or enhance their effects. Unlike alternative medicine—which may be used in place of conventional methods—complementary medicine is typically applied alongside conventional therapies.

The Questionnaire

2. **1. CAPTCHA:**
   Please solve the following arithmetic problem to prove you are not a robot:
   eight plus three equals
   (Free-response field)
3. **Where is your main residence?**
   - Postal Code
   - City
   - Country
4. **How old are you?**
   (Free-response field)
5. **Which gender do you identify with?**

- Female
- Male
- Diverse
- Prefer not to say

6. **What is your highest level of education?**
    - Primary school or below / Lower secondary school
    - Polytechnical school
    - Vocational school (e.g., HASCH)
    - Apprenticeship, vocational school
    - University entrance exam (Studienberechtigungsprüfung)
    - Professional maturity exam / Evening school diploma
    - General secondary school (AHS) with Matura
    - Technical or commercial secondary school (BHS, e.g., HTL, HAK, HBLA, etc.) with Matura
    - University-affiliated institution or college
    - Bachelor‘s
    - Magister/Master’s/Diploma Engineer, University of Applied Sciences, Medicine
    - Doctorate/PhD
    - Other school
    - I don’t know

7. **What is your approximate monthly net income?**
   (This refers to the amount you receive from your employer.)

   - I do not have a personal income
   - Less than €250
   - €250 to €499
   - €500 to €999
   - €1,000 to €1,499
   - €1,500 to €1,999
   - €2,000 to €2,499
   - €2,500 to €2,999
   - €3,000 to €3,499
   - €3,500 to €3,999
   - €4,000 or more
   - Prefer not to say

2. **Imagine that you suffer from “chronic migraines.” This condition leads to frequent and intense headache attacks that significantly affect your quality of life. There are conventional treatments (often called “school medicine”), such as medications, as well as various alternative and complementary treatment approaches. Which of these treatment options would you be most likely to use?**

   - Exclusively conventional medicine – no alternative or complementary approaches
   - Primarily conventional medicine, with alternative/complementary approaches as an add-on
   - A combination of both conventional and alternative/complementary medicine
   - Primarily alternative/complementary medicine, with conventional medicine used only in exceptional cases

- Exclusively alternative/complementary medicine – no conventional medicine

3. **Have you heard of anthroposophic medicine?**
    - Yes, and I can explain what anthroposophic medicine means
    - Yes, I am familiar with the term but cannot sufficiently explain it
    - No, I have never heard of it
    - I don't know

If you answer "No" or "I don't know":
Briefing: Anthroposophic medicine is a part of complementary medicine. It is based on the teachings of anthroposophy as developed by Dr. Rudolf Steiner. Its goal is to explore the physical, soul-related, and spiritual aspects of the human being and their connection with nature and the cosmos. It is intended as a supplement to conventional medicine.

4. **To what extent do you agree with the following statement: "Anthroposophic medicine is a scientifically based treatment method."**
    - Strongly agree
    - Partially agree
    - Slightly agree
    - Do not agree at all
    - I don't know

11. **How frequently do you use anthroposophic medicine?**
    - Never
    - Tried it once
    - Rarely (once a year or less)
    - Occasionally (several times a year)

- Regularly (monthly or more often)

12. **To what extent do you agree with the following statement: "Doctors who are trained in anthroposophic medicine and use it in their treatment of patients are more competent than those who do not offer it."**
    - Strongly agree
    - Partially agree
    - Slightly agree
    - Do not agree at all
    - I don't know

13. **In the past 12 months, have you consciously visited a doctor who offers alternative or complementary medical treatments (e.g., acupuncture, traditional Chinese medicine, homeopathy, anthroposophic medicine, etc.)?**
    - Yes
    - No
    - I don't know
    - In the past 12 months, have you visited a doctor who offers exclusively conventional medicine?
    - Yes
    - No
    - I don't know

15. **Do you regularly use preventive medical check-ups (e.g., mammography, PAP smear, colonoscopy from age 50, general health examinations), or do you plan to in the future?**
    - Yes, I have used preventive check-ups in the past and plan to continue doing so in the future
    - Yes, I have used preventive check-ups in the past, but I do not plan to in the future

- Yes, I plan to use preventive check-ups in the future, although I have not yet done so
- No, I have never used and do not plan to use preventive check-ups

16. If you answered "Yes" to question 15, which check-ups?
(Free-response field or select multiple options such as mammography, PAP smear, colonoscopy from age 50, general health examination)

17. Do you vaccinate yourself or your children according to national vaccination recommendations?

- Strongly agree
- Partially agree
- Slightly agree
- Do not agree at all
- I don't know

18. Does the term "Evidence-Based Medicine (EBM)" mean anything to you?

- Yes, and I can explain what EBM means
- Yes, I am familiar with the term but cannot sufficiently explain it
- No, I have never heard of it
- I don't know

19. To what extent do you agree with the following statement: "A scientific basis derived from studies is important to me when selecting a medical therapy."

- Strongly agree
- Partially agree
- Slightly agree
- Do not agree at all

- I don't know

20. To what extent do you agree with the following statement: "If a treatment has been used for a long time and treatment successes have been reported, I would trust that treatment more than one that has a scientific basis."

- Strongly agree
- Partially agree
- Slightly agree
- Do not agree at all
- I don't know

21. If national parliamentary elections were held next Sunday, which party would you vote for?

- FPÖ – Freedom Party of Austria
- ÖVP – People's Party
- SPÖ – Social Democratic Party of Austria
- NEOS – The New Austria and Liberal Forum
- Die Grünen – The Green Alternative
- KPÖ – Communist Party of Austria
- Bier – The Beer Party
- LMP – Madeleine Petrovic List
- NONE – None of the above
- MFG – Österreich – People, Freedom, Fundamental Rights
- GAZA – Vote Against Genocide

- BGE – The Yellow Party
- Other party: (please specify)
- I would cast an invalid vote
- I would not vote at all
- I don’t know

22. Which services should be covered by the health insurance, and which should not?

- Conventional medicine: e.g., medications, surgeries, etc.
- Alternative medicine: e.g., acupuncture, homeopathy, herbal medicine, etc.
- Complementary medicine: e.g., anthroposophic medicine, etc.
- I don’t know

23. Reflecting on your past contact with doctors and healthcare professionals practicing conventional medicine: Did you generally feel taken seriously, or that decisions were made over your head?

- I was almost always taken seriously
- I was mostly taken seriously
- Sometimes yes, sometimes no
- I was mostly not taken seriously
- I was almost never taken seriously
- I don’t know

24. Are you currently suffering from, or have you suffered from in the last 3 years, one or more of the following conditions?

- Infectious diseases

- Cancer
- Blood/immune system disorders
- Nutritional and metabolic diseases
- Nervous system disorders
- Mental health disorders
- Eye diseases
- Ear diseases
- Cardiovascular diseases
- Respiratory diseases
- Gastrointestinal diseases
- Skin diseases
- Diseases of muscles, bones, and joints
- Urogenital diseases (urinary and reproductive organs)
- Congenital diseases and malformations
- Injuries
- Other condition(s)
- No known condition
- I don’t know
- I prefer not to answer

25. How much money did you spend last year on private doctors for conventional medical treatments?
(Note: Private doctors' services are not billed through the health insurance)

- €0
- €1 to €99
- €100 to €249
- €250 to €499
- €500 to €749
- €750 to €999
- €1,000 or more
- Prefer not to say

26. How much money did you spend last year on complementary or alternative medical care (e.g., acupuncture, traditional Chinese medicine, homeopathy, anthroposophic medicine, etc.)?

- €0
- €1 to €99
- €100 to €249
- €250 to €499
- €500 to €749
- €750 to €999
- €1,000 or more
- Prefer not to say

27. How often do you engage in sports (e.g., endurance training, strength training, etc.) per week?

- No physical activity
- Less than 1 hour per week
- Regularly, 1–2 hours per week
- Regularly, 2–4 hours per week
- Regularly, more than 4 hours per week
- I cannot say

28. Which of the following best describes your dietary habits?

- Very frequently: fast food, snacks, sweet pastries, processed meat products (e.g., sausage), red meat, full-fat dairy products, sweetened beverages, few freshly prepared meals, little fruit and vegetables
- Frequently: fast food, snacks, sweet pastries, processed meat products (e.g., sausage), red meat, full-fat dairy products, sweetened beverages; sometimes freshly prepared meals, occasional fruit and vegetables
- Regularly: plant-based and freshly prepared meals, legumes (e.g., lentils, beans), frequent fruit and vegetables, whole grain products, fish, healthy fats (e.g., nuts, avocado); occasional snacks or sweet pastries
- Predominantly: plant-based and freshly prepared meals, legumes (e.g., lentils, beans), plenty of fruit and vegetables, whole grain products, fish, healthy fats (e.g., nuts, avocado); hardly any snacks or sweet pastries

29. Overall, do you feel satisfied with your life?

- Extremely
- Quite a bit
- Moderately
- A little
- Not at all

30. Do you look to the future with optimism?

- Extremely
- Quite a bit
- Moderately
- A little
- Not at all

31. Do you tend to worry or ruminate a lot?

- Extremely
- Quite a bit
- Moderately
- A little
- Not at all

32. Do your spiritual or religious beliefs help you make sense of life's difficulties?

- Extremely
- Quite a bit
- Moderately
- A little
- Not at all

33. I feel that I have control over my own health.

- Strongly agree

- Partially agree
- Neutral
- Slightly disagree
- Strongly disagree

34. Whether I am healthy or ill is largely a matter of luck.

- Strongly agree
- Partially agree
- Neutral
- Slightly disagree
- Strongly disagree

35. When I have been disturbed, emotionally aroused, or thrown off balance by something or someone, I tell myself, “Everything will be alright.”

- Strongly agree
- Partially agree
- Neutral
- Slightly disagree
- Strongly disagree

36. When I have been disturbed, emotionally aroused, or thrown off balance by something or someone, I take measures to eliminate the cause.

- Strongly agree
- Partially agree
- Neutral

- Slightly disagree
- Strongly disagree

37. When I become ill, it is largely my own fault.

- Strongly agree
- Partially agree
- Neutral
- Slightly disagree
- Strongly disagree

38. For every problem, I can find a solution.

- Strongly agree
- Partially agree
- Neutral
- Slightly disagree
- Strongly disagree

39. Even in the face of unexpected events, I believe I can cope well.

- Strongly agree
- Partially agree
- Neutral
- Slightly disagree
- Strongly disagree

40. I am willing to adjust my daily life to promote my long-term health, even if it is time-consuming.

- Strongly agree
- Partially agree
- Neutral
- Slightly disagree
- Strongly disagree

41. I would refrain from consuming indulgences (such as alcohol, tobacco, sugar) in order to improve my long-term health.

- Strongly agree
- Partially agree
- Neutral
- Slightly disagree
- Strongly disagree

42. The sooner we all adopt similar values and beliefs, the better our society will be.

- Strongly agree
- Partially agree
- Neutral
- Slightly disagree
- Strongly disagree
- I don't know

43. Experts who cannot provide a clear answer probably do not know much.

- Strongly agree
- Partially agree
- Neutral
- Slightly disagree
- Strongly disagree

44. We need strong leaders so that we can live securely in society.

- Strongly agree
- Partially agree
- Neutral
- Slightly disagree
- Strongly disagree

45. Established ways of behaving should not be questioned.

- Strongly agree
- Partially agree
- Neutral
- Slightly disagree
- Strongly disagree

End of Survey
