# Supplementary material for: Authoritarian attitudes and the perceived scientific legitimacy of anthroposophic medicine: A survey of attitudes on complementary and alternative medicine in Austria
Source: PLoS One. 2026 Jun 17;21(6):e0348672. doi: 10.1371/journal.pone.0348672 (PMC13274894; doi:10.1371/journal.pone.0348672)
Supplement: S2 File — Explanation of political parties. (PDF) [file pone.0348672.s007.pdf]

Supplement 7: Explanation of political parties

Political Party	Name in English	Description
Grüne	The Greens	A progressive party emphasizing environmental protection, sustainability, and social justice.
SPÖ	Social Democratic Party	A centre-left party focusing on social welfare, equality, and labour rights.
NEOS	NEOS—The New Austria and Liberal Forum	A liberal reformist party advocating civil liberties, free markets, and modernization.
KPÖ	Communist Party of Austria	A left-wing party based on communist principles, prioritizing workers' rights and social equity.
ÖVP	Austrian People's Party	A centre-right party promoting conservative values, economic liberalism, and national identity.
FPÖ	Freedom Party of Austria	A right-wing populist party known for its nationalist stance and skepticism toward immigration.
Bier Die Bierpartei	Beer—The Beer Party	A satirical or novelty party that uses humour to critique politics and engage disenchanted voters.
GAZA	GAZA	A minor party with alternative political views that challenges mainstream political discourse.
LMP	List of Madeleine Petrovic	A small progressive party focused on modern reformist policies and innovation in governance.
