# Supplementary material for: Authoritarian attitudes and the perceived scientific legitimacy of anthroposophic medicine: A survey of attitudes on complementary and alternative medicine in Austria
Source: PLoS One. 2026 Jun 17;21(6):e0348672. doi: 10.1371/journal.pone.0348672 (PMC13274894; doi:10.1371/journal.pone.0348672)
Supplement: Attachment — Submitted filename: Response_to_Reviewers_final.docx [file pone.0348672.s010.docx]

**Response to Reviewers**

PONE-D-25-42101

*Authoritarian Attitudes and the Perceived Scientific Legitimacy of Anthroposophic Medicine: A Survey of Attitudes on Complementary and Alternative Medicine in Austria*

Dear Editor and Reviewers,

We sincerely thank both reviewers for their thorough, constructive, and intellectually generous engagement with our manuscript. Their feedback has significantly strengthened the paper. Below, we provide a point-by-point response to all comments, detailing the changes made in the revised manuscript. Reviewer comments are presented in grey italics, followed by our responses.

Minor formatting corrections (p-value standardization, symbol corrections, reference formatting, typographical fixes) were applied throughout and are not individually tracked. All substantive revisions are reflected in the tracked-changes version of the manuscript.

We trust that these revisions address the concerns raised and strengthen the manuscript. We are grateful for the opportunity to revise and resubmit.

Sincerely,

The authors

# **Journal Requirements**

## **1. Style Requirements**

*Please ensure that your manuscript meets PLOS ONE’s style requirements, including those for file naming.*

Done. The manuscript has been reformatted to comply with PLOS ONE style requirements. Reference formatting has been checked and corrected throughout. File naming conventions have been updated accordingly.

## **2. Data Sharing / Data Availability Statement**

*PLOS only allows data to be available upon request if there are legal or ethical restrictions on sharing data publicly.*

The dataset underlying the results of this study can be obtained from the corresponding author upon reasonable request and will be made available for further analyses. We are currently clarifying institutional requirements regarding data sharing and, if permitted, will deposit the dataset in a public repository in accordance with PLOS data availability policies.

## **3. Identifying Data in Supporting Information**

*There is identifying data in the Supporting Information file. Please remove or anonymize all personal information.*

We have thoroughly anonymized the Supporting Information file. All columns containing personal or potentially identifying information (including any hidden columns) have been permanently removed. The anonymized supplements have been re-uploaded.

## **4. Figures Upload**

*Please upload a copy of Figures 1–6 as separate files.*

Done. All six figures have been uploaded as separate high-resolution files.

## **5. Copyrighted Map Image (Figure 2)**

*Figure 2 contains map images which may be copyrighted. You must either present written permission or replace with a public domain alternative.*

To comply with the journal’s copyright and data policy, we replaced the figure with a newly generated map created in R using openly licensed geospatial data. The base map is derived from Statistics Austria Open Data (municipality boundaries; CC BY 4.0) and postal code locations were obtained from the GeoNames postal code dataset (CC BY). The revised figure relies exclusively on openly licensed data sources and no longer contains copyrighted map tiles.

## **6. Table 1 Reference Missing**

*Please ensure that you refer to Table 1 in your text.*

Done. An explicit reference to Table 1 has been added in the Results section.

## **7. Supporting Information Captions**

*Please include captions for your Supporting Information files at the end of your manuscript.*

Done. Captions for all Supporting Information files (S1–S7) have been added at the end of the manuscript.

## **8. Recommended Citations**

*If the reviewer comments include a recommendation to cite specific previously published works, please review and evaluate these publications.*

Noted. No reviewer made specific citation recommendations. We have added one new reference (Bloor, 1991) in response to Reviewer 2’s invitation for epistemological reflexivity (Comment 2.22).

# **Reviewer #1**

*We thank Reviewer 1 for their positive assessment and detailed suggestions for improvement.*

## **Comment 1.1 – Title**

*The study title is informative and easy to understand, but it is too detailed and looks like a results section.*

We agree. The title has been revised to: “Authoritarian Attitudes and the Perceived Scientific Legitimacy of Anthroposophic Medicine: A Survey of Attitudes on Complementary and Alternative Medicine in Austria.”

## **Comment 1.2 – Introduction**

*The introduction has too much length and density. The tone occasionally veers toward polemical criticism. The current study’s justification comes late.*

We have substantially condensed and restructured the Introduction from nine to six paragraphs. The revised version: (a) explains AM’s popularity, including institutional integration, perceived holistic benefits, and cultural visibility (Waldorf, biodynamic agriculture, lifestyle products); (b) defines Steiner’s “spiritual science” (Geisteswissenschaft) and specifies which epistemological principles are incompatible with scientific methodology; (c) removed the underdeveloped climate change reference; (d) sharpened the Nazi/AM passage to foreground epistemological parallels with primary source citations for Steiner’s racist and antisemitic writings; and (e) adopted a consistently scholarly-neutral tone.

## **Comment 1.3 – Methods**

*Online convenience sampling reduces representativeness; no pre-registration or power analysis; no reliability coefficients; post-stratification weighting may not sufficiently account for demographic disparities; more information about data protection and informed consent is necessary.*

**Sampling limitations:** We clarified the sampling procedure and its implications. The Methods section now explicitly states that participants were recruited through online convenience sampling. The Strengths and Weaknesses section discusses the consequences, including potential self-selection bias and limited representativeness.

**Ethics, data protection, and informed consent:** We expanded the description of ethical procedures and data protection. The manuscript now clarifies that participation was voluntary and anonymous, that informed consent was obtained before participation, and that no directly identifying personal data were collected.

**Reliability coefficients:** Cronbach’s α was calculated for all multi-item measures. Reliability varied across scales: WHOQOL (0.52), Self-Efficacy (0.64), Stress Management (0.59), MSTAT-II (0.43), KSA-3 (0.49), MHLC composites (0.30/0.32). The adapted health locus of control composites showed low internal consistency and were therefore interpreted with caution.

**Post-stratification:** We expanded the explanation of the weighting procedure. Post-stratification weights were calculated as the ratio between population proportions (derived from Austrian National Council election results) and observed sample proportions for each political category. The limitations of this procedure are acknowledged.

**Pre-registration and power analysis:** The manuscript now explicitly acknowledges that the study was not preregistered and no formal a priori power analysis was conducted. The analyses are accordingly interpreted as exploratory.

**Measurement limitations:** We added a note that several abbreviated or adapted multi-item measures showed only low to moderate internal consistency, which may have attenuated associations.

## **Comment 1.4 – Results**

*The results section is unduly descriptive. Tables and figures are not adequately interpreted. A stronger focus on effect sizes is needed.*

We substantially revised the Results section. It is now organized along the research question clusters. Demographic composition has been moved to Methods. Single-item results have been removed. For all regression analyses, we now report regression coefficients (B), standard errors (SE), t-values, p-values, 95% confidence intervals, and model fit indices (R²). Four regression tables have been added to the manuscript. Effect sizes are reported where appropriate.

## **Comment 1.5 – Discussion**

*The discussion is excessively verbose and occasionally repetitious. There is a risk of overgeneralization beyond the correlational nature of the data.*

The Discussion has been substantially tightened. All causal language (“why”) has been replaced with correlational framing (“what” and “how”). The key finding—the association between authoritarian orientation and perceived scientific validity of AM—now receives expanded treatment, including a new paragraph on the dissociation between epistemic judgment and behavioral choice. A reflexivity paragraph has been added. The term “interdisciplinary” has been replaced with “socio-psychological inquiry” where referring to this study’s methods. Three redundant closing paragraphs have been removed.

## **Comment 1.6 – References**

*Please modify and check reference styles per the journal guidelines.*

Done. All references have been reformatted to PLOS ONE citation style.

# **Reviewer #2**

*We are grateful for Reviewer 2’s enthusiastic and incisive engagement with our work. Their comments—particularly regarding epistemological reflexivity and the authoritarianism finding—have meaningfully deepened the revised manuscript.*

## **Comment 2.1 – Raw Data Availability**

*Please ensure all raw data is made publicly available.*

See our response to Journal Requirement 2. The anonymized dataset can be obtained from the corresponding author upon reasonable request. We are clarifying institutional requirements for public repository deposit.

## **Comment 2.2 – General Assessment**

*Ultimately an incredibly fun manuscript to review. I am excited to see it get cleaned up to be even stronger.*

We sincerely appreciate this generous assessment and have aimed to honor the reviewer’s comprehensive engagement by addressing each point carefully.

## **Comment 2.3 – AM Popularity, Benefits, and Conceptual Definitions**

*It is unclear why or how this form of medicine has become popularized. Concepts are introduced without definition or explanation.*

The revised Introduction now explains AM’s popularity through institutional integration, holistic framing, and broader cultural visibility; defines Steiner’s Geisteswissenschaft as supersensible cognition cultivated through meditative practice; specifies the epistemological incompatibilities; and names the core metaphysical commitments (etheric bodies, formative forces, karmic processes). The climate change reference has been removed.

## **Comment 2.4 – Nazi/AM Connection**

*Clarify the epistemological parallelisms between AM and Nazism. Which antisemitic and racist beliefs were present in Steiner’s writings?*

The passage now foregrounds structural-epistemological parallels: hierarchical cosmology, mystified epistemic authority, resistance to critical-rational inquiry. Steiner’s racist and antisemitic writings are cited with primary sources [24, 25]. The Heydrich quotation has been removed; the Adorno/Benjamin/Bloch framework has been retained and tightened.

## **Comment 2.5 – CAM Acronym**

*You define the CAM acronym in the abstract but not in the introduction.*

Done. “Complementary and alternative medicine (CAM)” is now introduced at first use in the main text.

## **Comment 2.6 – Cronbach’s Alpha**

*Please perform and report Cronbach’s Alphas for each measure used.*

Cronbach’s α has been calculated for all multi-item measures and is reported in the Methods section. See our response to Comment 1.3 for the values.

## **Comment 2.7 – Sociodemographic “Scores” and Statistical Tests**

*What are the “scores” you are referencing? Age is continuous while gender is categorical.*

We revised the manuscript to clearly distinguish between continuous and categorical variables. The Methods section now specifies independent-samples t-tests for two-group comparisons of continuous variables, one-way ANOVA for more than two groups, Mann-Whitney U or Kruskal-Wallis tests for non-normally distributed variables, and chi-square or Fisher’s exact tests for categorical variables. Sociodemographic variables are treated according to their measurement level.

## **Comment 2.8 – P-Value Standardization**

*Please standardize your reporting of p-values to not go below p < .001.*

Done. All p-values standardized throughout.

## **Comment 2.9 – Explicit Test Labels and Statistics**

*Please make clear which test was performed and ensure all necessary values are reported.*

Each statistical test is now explicitly named. Regression results include B, SE, t-values, 95% CIs, p-values, and R². Demographic table footnotes specify whether chi-square or Fisher’s exact tests were applied.

## **Comment 2.10 – Demographics in Methods**

*Description of the demographic composition would fit more neatly within the method section.*

Agreed. Demographic description has been moved to Methods.

## **Comment 2.11 – Forest Plots**

*The forest plots do not yet help illustrate your results. It is unclear which independent variables are included.*

Forest plots have been revised with clearer labeling. The label in Figure 4 has been corrected from “Fascist tendencies” to “Authoritarian Attitudes (KSA-3).”

## **Comment 2.12 – Remove Single-Item Results**

*Please remove any results pertaining to specific items.*

Agreed. All results based on individual survey items have been removed. Only scale-level findings are reported.

## **Comment 2.13 – Post-Stratification Justification**

*Please justify your inclusion of post-stratification techniques.*

The Methods section now includes a dedicated justification. The distributional skew in political party affiliation indicated the sample was not representative. Population targets were derived from official Austrian National Council election results. Weights were calculated as the ratio between population and sample proportions per political category. The procedure and its limitations are now described in detail.

## **Comment 2.14 – Results Organization**

*Consider organizing based on the presented clusters of research questions.*

Done. Results reorganized along the research question clusters.

## **Comment 2.15 – Duplicate Sentence**

*The first sentence in the discussion section repeats itself.*

Done. Corrected.

## **Comment 2.16 – Causal Language**

*Be careful introducing questions of “why.”*

All instances of “why” in the Discussion have been replaced with “what” and “how.”

## **Comment 2.17 – “Interdisciplinary”**

*I do not think “interdisciplinary” is an apt word to describe this study.*

Agreed. References to this study as “interdisciplinary” have been replaced with “socio-psychological inquiry.”

## **Comment 2.18 – “Cross-Sectional” Framing**

*I do not find it appropriate to call this study “cross-sectional.”*

We have removed “cross-sectional” as a design descriptor from the Discussion. Age is positioned as a covariate rather than a design element.

## **Comment 2.19 – “Could Be Determined as Significant”**

*The phrasing that these variables “could be” significant is vague.*

Replaced with precise statistical reporting: “In the unweighted model, low ambiguity tolerance (B = 1.66, p = .013), prioritization of health optimization (B = 1.78, p < .001), and authoritarian attitudes (B = 1.72, p = .015) were each significantly associated with greater CAM endorsement.”

## **Comment 2.20 – CAM Preference and Lower Stress Tolerance**

*I would like to see a stronger interpretation of this association.*

The revised Discussion considers several mechanisms: CAM’s longer consultations and participatory therapeutic relationship may offer greater patient agency; the holistic framing may reduce situational distress; conventional medicine’s probabilistic reasoning may be particularly aversive for individuals with low stress tolerance. These are framed as interpretive hypotheses requiring further investigation.

## **Comment 2.21 – Non-Significant Authoritarianism Result**

*Please note any potential statistical or methodological limitations. Maybe there are stronger ways to measure authoritarianism.*

A dedicated paragraph addresses: (a) the KSA-3’s limitations as a brief screening instrument; (b) shared variance absorbed by post-stratification with political affiliation; (c) whether authoritarianism requires updated operationalization; and (d) the critical distinction that non-significance pertains to CAM preference, while the association with perceived scientific validity of AM remained robust (p < .001).

## **Comment 2.22 – Reflexivity Paragraph**

*I think it’s critically necessary to recognize that you are performing research using positivist methods that reproduce this very same episteme.*

A reflexivity paragraph has been added drawing on the sociology of scientific knowledge (Bloor, 1991 [42]). We acknowledge that positivist-empirical methods operate within the same regime of epistemic authority whose dynamics we investigate. The symmetry principle is applied reflexively. We conclude that the study is situated as an intervention within, rather than an adjudication upon, the field of competing knowledge claims.

## **Comment 2.23 – Authoritarianism and Scientific Validity: Go Deeper**

*The key finding appears to be the association between authoritarianism and perceived scientific validity. This should fill up a larger portion of your discussion.*

The revised Discussion gives this finding substantially expanded treatment: (a) the dissociation between robust association with perceived scientific validity (p < .001) and non-significant link with actual AM use (p = 0.536); (b) “scientific validity” as a marker of epistemic trust shaped by perceived source authority rather than evidence engagement; and (c) connections to the Adorno/Frenkel-Brunswik tradition and its contemporary relevance.
