# Supplementary material for: Authoritarian attitudes and the perceived scientific legitimacy of anthroposophic medicine: A survey of attitudes on complementary and alternative medicine in Austria
Source: PLoS One. 2026 Jun 17;21(6):e0348672. doi: 10.1371/journal.pone.0348672 (PMC13274894; doi:10.1371/journal.pone.0348672)
Supplement: Peer review history

# Peer review: Decision Letter 0

**DOI:** 10.1371/journal.pone.0348672.r001

**Authors:** Jiajia Ye

1 Feb 2026

PONE-D-25-42101

Socioeconomic and Psychological Determinants of Anthroposophic Medicine Use: A Cross-Sectional Survey Highlighting Statistically Relevant Associations with Age, Gender, Perceived Health Control, and Ambiguity Tolerance

PLOS One

Dear Dr.  Sitte,

Thank you for submitting your manuscript to PLOS ONE. After careful consideration, we feel that it has merit but does not fully meet PLOS ONE’s publication criteria as it currently stands. Therefore, we invite you to submit a revised version of the manuscript that addresses the points raised during the review process.

Please submit your revised manuscript by Mar 18 2026 11:59PM. If you will need more time than this to complete your revisions, please reply to this message or contact the journal office at plosone@plos.org. When you're ready to submit your revision, log on to https://www.editorialmanager.com/pone/ and select the 'Submissions Needing Revision' folder to locate your manuscript file.

Please include the following items when submitting your revised manuscript:

- A letter that responds to each point raised by the academic editor and reviewer(s). You should upload this letter as a separate file labeled 'Response to Reviewers'.
- A marked-up copy of your manuscript that highlights changes made to the original version. You should upload this as a separate file labeled 'Revised Manuscript with Track Changes'.
- An unmarked version of your revised paper without tracked changes. You should upload this as a separate file labeled 'Manuscript'.

If you would like to make changes to your financial disclosure, please include your updated statement in your cover letter. Guidelines for resubmitting your figure files are available below the reviewer comments at the end of this letter.

If applicable, we recommend that you deposit your laboratory protocols in protocols.io to enhance the reproducibility of your results. Protocols.io assigns your protocol its own identifier (DOI) so that it can be cited independently in the future. For instructions see: https://journals.plos.org/plosone/s/submission-guidelines#loc-laboratory-protocols. Additionally, PLOS ONE offers an option for publishing peer-reviewed Lab Protocol articles, which describe protocols hosted on protocols.io. Read more information on sharing protocols at https://plos.org/protocols?utm_medium=editorial-email&utm_source=authorletters&utm_campaign=protocols.

We look forward to receiving your revised manuscript.

Kind regards,

Jiajia Ye

Guest Editor

PLOS One

Journal Requirements:

When submitting your revision, we need you to address these additional requirements.

1.Please ensure that your manuscript meets PLOS ONE's style requirements, including those for file naming. The PLOS ONE style templates can be found at

https://journals.plos.org/plosone/s/file?id=wjVg/PLOSOne_formatting_sample_main_body.pdf and

https://journals.plos.org/plosone/s/file?id=ba62/PLOSOne_formatting_sample_title_authors_affiliations.pdf

2. We note that you have indicated that there are restrictions to data sharing for this study. PLOS only allows data to be available upon request if there are legal or ethical restrictions on sharing data publicly. For more information on unacceptable data access restrictions, please see http://journals.plos.org/plosone/s/data-availability#loc-unacceptable-data-access-restrictions.

Before we proceed with your manuscript, please address the following prompts:

a) If there are ethical or legal restrictions on sharing a de-identified data set, please explain them in detail (e.g., data contain potentially identifying or sensitive patient information, data are owned by a third-party organization, etc.) and who has imposed them (e.g., a Research Ethics Committee or Institutional Review Board, etc.). Please also provide contact information for a data access committee, ethics committee, or other institutional body to which data requests may be sent.

b) If there are no restrictions, please upload the minimal anonymized data set necessary to replicate your study findings to a stable, public repository and provide us with the relevant URLs, DOIs, or accession numbers. For a list of recommended repositories, please see

https://journals.plos.org/plosone/s/recommended-repositories. You also have the option of uploading the data as Supporting Information files, but we would recommend depositing data directly to a data repository if possible.

We will update your Data Availability statement on your behalf to reflect the information you provide.

3. We note that there is identifying data in the Supporting Information file “Supplements_2025.04.08_NR.pdf”. Due to the inclusion of these potentially identifying data, we have removed this file from your file inventory. Prior to sharing human research participant data, authors should consult with an ethics committee to ensure data are shared in accordance with participant consent and all applicable local laws.

Data sharing should never compromise participant privacy. It is therefore not appropriate to publicly share personally identifiable data on human research participants. The following are examples of data that should not be shared:

-Name, initials, physical address

-Ages more specific than whole numbers

-Internet protocol (IP) address

-Specific dates (birth dates, death dates, examination dates, etc.)

-Contact information such as phone number or email address

-Location data

-ID numbers that seem specific (long numbers, include initials, titled “Hospital ID”) rather than random (small numbers in numerical order)

Data that are not directly identifying may also be inappropriate to share, as in combination they can become identifying. For example, data collected from a small group of participants, vulnerable populations, or private groups should not be shared if they involve indirect identifiers (such as sex, ethnicity, location, etc.) that may risk the identification of study participants.

Additional guidance on preparing raw data for publication can be found in our Data Policy (https://journals.plos.org/plosone/s/data-availability#loc-human-research-participant-data-and-other-sensitive-data) and in the following article: http://www.bmj.com/content/340/bmj.c181.long.

Please remove or anonymize all personal information (<specific identifying information in file to be removed>), ensure that the data shared are in accordance with participant consent, and re-upload a fully anonymized data set. Please note that spreadsheet columns with personal information must be removed and not hidden as all hidden columns will appear in the published file.

4. Please upload a copy of Figure 1, 2, 3, 4, 5 and 6, to which you refer in your text on page 25. If the figure is no longer to be included as part of the submission please remove all reference to it within the text.

5. We note that Figure 2 in your submission contain map images which may be copyrighted. All PLOS content is published under the Creative Commons Attribution License (CC BY 4.0), which means that the manuscript, images, and Supporting Information files will be freely available online, and any third party is permitted to access, download, copy, distribute, and use these materials in any way, even commercially, with proper attribution. For these reasons, we cannot publish previously copyrighted maps or satellite images created using proprietary data, such as Google software (Google Maps, Street View, and Earth). For more information, see our copyright guidelines: http://journals.plos.org/plosone/s/licenses-and-copyright.

We require you to either (1) present written permission from the copyright holder to publish these figures specifically under the CC BY 4.0 license, or (2) remove the figures from your submission:

1. You may seek permission from the original copyright holder of Figure 2 to publish the content specifically under the CC BY 4.0 license.

We recommend that you contact the original copyright holder with the Content Permission Form (http://journals.plos.org/plosone/s/file?id=7c09/content-permission-form.pdf) and the following text:

“I request permission for the open-access journal PLOS ONE to publish XXX under the Creative Commons Attribution License (CCAL) CC BY 4.0 (http://creativecommons.org/licenses/by/4.0/). Please be aware that this license allows unrestricted use and distribution, even commercially, by third parties. Please reply and provide explicit written permission to publish XXX under a CC BY license and complete the attached form.”

Please upload the completed Content Permission Form or other proof of granted permissions as an "Other" file with your submission.

In the figure caption of the copyrighted figure, please include the following text: “Reprinted from [ref] under a CC BY license, with permission from [name of publisher], original copyright [original copyright year].”

2. If you are unable to obtain permission from the original copyright holder to publish these figures under the CC BY 4.0 license or if the copyright holder’s requirements are incompatible with the CC BY 4.0 license, please either i) remove the figure or ii) supply a replacement figure that complies with the CC BY 4.0 license. Please check copyright information on all replacement figures and update the figure caption with source information. If applicable, please specify in the figure caption text when a figure is similar but not identical to the original image and is therefore for illustrative purposes only.

The following resources for replacing copyrighted map figures may be helpful:

USGS National Map Viewer (public domain): http://viewer.nationalmap.gov/viewer/

The Gateway to Astronaut Photography of Earth (public domain): http://eol.jsc.nasa.gov/sseop/clickmap/

Maps at the CIA (public domain): https://www.cia.gov/library/publications/the-world-factbook/index.html and https://www.cia.gov/library/publications/cia-maps-publications/index.html

NASA Earth Observatory (public domain): http://earthobservatory.nasa.gov/

Landsat: http://landsat.visibleearth.nasa.gov/

USGS EROS (Earth Resources Observatory and Science (EROS) Center) (public domain): http://eros.usgs.gov/#

Natural Earth (public domain): http://www.naturalearthdata.com/

6. We note you have included a table to which you do not refer in the text of your manuscript. Please ensure that you refer to Table 1 in your text; if accepted, production will need this reference to link the reader to the Table.

7. Please include captions for your Supporting Information files at the end of your manuscript, and update any in-text citations to match accordingly. Please see our Supporting Information guidelines for more information: http://journals.plos.org/plosone/s/supporting-information.

8. If the reviewer comments include a recommendation to cite specific previously published works, please review and evaluate these publications to determine whether they are relevant and should be cited. There is no requirement to cite these works unless the editor has indicated otherwise.

[Note: HTML markup is below. Please do not edit.]

Reviewer's Responses to Questions

**Comments to the Author**

1. Is the manuscript technically sound, and do the data support the conclusions?

The manuscript must describe a technically sound piece of scientific research with data that supports the conclusions. Experiments must have been conducted rigorously, with appropriate controls, replication, and sample sizes. The conclusions must be drawn appropriately based on the data presented.

Reviewer #1: Yes

Reviewer #2: Yes

**********

2. Has the statistical analysis been performed appropriately and rigorously?

Reviewer #1: Yes

Reviewer #2: Yes

**********

3. Have the authors made all data underlying the findings in their manuscript fully available?

The PLOS Data policy requires authors to make all data underlying the findings described in their manuscript fully available without restriction, with rare exception (please refer to the Data Availability Statement in the manuscript PDF file). The data should be provided as part of the manuscript or its supporting information, or deposited to a public repository. For example, in addition to summary statistics, the data points behind means, medians and variance measures should be available. If there are restrictions on publicly sharing data—e.g. participant privacy or use of data from a third party—those must be specified.

Reviewer #1: Yes

Reviewer #2: No

**********

4. Is the manuscript presented in an intelligible fashion and written in standard English?

PLOS ONE does not copyedit accepted manuscripts, so the language in submitted articles must be clear, correct, and unambiguous. Any typographical or grammatical errors should be corrected at revision, so please note any specific errors here.

Reviewer #1: Yes

Reviewer #2: Yes

**********

5. Review Comments to the Author

Please use the space provided to explain your answers to the questions above. You may also include additional comments for the author, including concerns about dual publication, research ethics, or publication ethics. (Please upload your review as an attachment if it exceeds 20,000 characters)

Reviewer #1: This study examines the prevalence, application, and perceived scientific validity of anthroposophic medicine (AM), a well-known type of complementary and alternative medicine (CAM). My observations are given below:

(1) The study title is okay. It is informative and easy to understand, but it is too detailed and looks like a results section. It lacks clarity regarding research gaps, a clear rationale, and theoretical underpinnings. The focus is mostly on statistical results, with little attention paid to conceptual contributions, limitations, and implications.

(2) The introduction needs revisions. It integrates the epistemological, sociocultural, and political aspects of anthroposophic medicine (AM) in a remarkably thorough historical and philosophical overview. However, it has too much length and density, which could make the main topic of the study difficult to see. Rather than remaining scholarly neutral, the tone occasionally veers toward polemical criticism. The current study's justification, which links AM use to psychological factors and socioeconomic determinants, comes late and doesn't provide a clear theoretical link to previous discussions. It is advised to be more succinct, conceptually coherent, and specifically aligned with research goals.

(3) The methods section is well-organized and includes pertinent statistical analyses, but there are a few issues. Online convenience sampling through news and social media platforms reduces representativeness and could lead to bias from self-selection. The robustness of inference is diminished when pre-registration and power analysis are not conducted. No reliability coefficients are provided for this sample, even though the measures used are known. Weighting after stratification based on voting patterns might not sufficiently account for demographic disparities. Lastly, even though the lack of official ethical approval is justified, more information about data protection and informed consent protocols is necessary.

(4) The results section is thorough but unduly descriptive, making it challenging to separate the main conclusions from the wealth of categorical and demographic information. The presentation lacks integration and narrative coherence connecting results to research objectives, despite the statistical rigor being apparent. Readers must deduce trends because tables and figures are not adequately interpreted. A few conclusions (e.g. G. psychological predictors and regression analyses) are fascinating, but they are not adequately contextualized or theoretically explained. Interpretability and scholarly value would be improved by a stronger focus on effect sizes, real-world applications, and more organized content.

(5) The discussion successfully connects psychological concepts with sociocultural and political aspects in an extensive, multidisciplinary, and intellectually stimulating manner. It exhibits contextual awareness and theoretical depth. The section, however, could use more organization and smoother transitions between empirical findings and theoretical implications, as it is excessively verbose and occasionally repetitious. There is a risk of overgeneralization when the interpretation goes beyond the correlational nature of the data. Stronger coherence, improved scientific rigor, and more accurate interpretation would result from a more thorough integration of earlier empirical research and a clearer connection between particular findings and psychological theory.

(6) Please modify and check reference styles aper the journal guidelines.

Reviewer #2: All necessary boxes have been checked except for the inclusion of your data. Please make sure that the dataset is made publicly available, either by uploading it to a digital database or by including it in your supplementary materials. All other comments can be seen organized in the attached spreadsheet for your convenience.

**********

6. PLOS authors have the option to publish the peer review history of their article (what does this mean?). If published, this will include your full peer review and any attached files.

If you choose “no”, your identity will remain anonymous but your review may still be made public.

[NOTE: If reviewer comments were submitted as an attachment file, they will be attached to this email and accessible via the submission site. Please log into your account, locate the manuscript record, and check for the action link "View Attachments". If this link does not appear, there are no attachment files.]

To ensure your figures meet our technical requirements, please review our figure guidelines: https://journals.plos.org/plosone/s/figures

You may also use PLOS’s free figure tool, NAAS, to help you prepare publication quality figures: https://journals.plos.org/plosone/s/figures#loc-tools-for-figure-preparation.

NAAS will assess whether your figures meet our technical requirements by comparing each figure against our figure specifications.

Attachment Submitted filename: peer review.xlsx

# Author comment: Author response to Decision Letter 1

**DOI:** 10.1371/journal.pone.0348672.r002

19 Mar 2026

Reviewer #1

We thank Reviewer 1 for their positive assessment and detailed suggestions for improvement.

Comment 1.1 – Title

The study title is informative and easy to understand, but it is too detailed and looks like a results section.

We agree. The title has been revised to: “Authoritarian Attitudes and the Perceived Scientific Legitimacy of Anthroposophic Medicine: A Survey of Attitudes on Complementary and Alternative Medicine in Austria.”

Comment 1.2 – Introduction

The introduction has too much length and density. The tone occasionally veers toward polemical criticism. The current study’s justification comes late.

We have substantially condensed and restructured the Introduction from nine to six paragraphs. The revised version: (a) explains AM’s popularity, including institutional integration, perceived holistic benefits, and cultural visibility (Waldorf, biodynamic agriculture, lifestyle products); (b) defines Steiner’s “spiritual science” (Geisteswissenschaft) and specifies which epistemological principles are incompatible with scientific methodology; (c) removed the underdeveloped climate change reference; (d) sharpened the Nazi/AM passage to foreground epistemological parallels with primary source citations for Steiner’s racist and antisemitic writings; and (e) adopted a consistently scholarly-neutral tone.

Comment 1.3 – Methods

Online convenience sampling reduces representativeness; no pre-registration or power analysis; no reliability coefficients; post-stratification weighting may not sufficiently account for demographic disparities; more information about data protection and informed consent is necessary.

Sampling limitations: We clarified the sampling procedure and its implications. The Methods section now explicitly states that participants were recruited through online convenience sampling. The Strengths and Weaknesses section discusses the consequences, including potential self-selection bias and limited representativeness.

Ethics, data protection, and informed consent: We expanded the description of ethical procedures and data protection. The manuscript now clarifies that participation was voluntary and anonymous, that informed consent was obtained before participation, and that no directly identifying personal data were collected.

Reliability coefficients: Cronbach’s α was calculated for all multi-item measures. Reliability varied across scales: WHOQOL (0.52), Self-Efficacy (0.64), Stress Management (0.59), MSTAT-II (0.43), KSA-3 (0.49), MHLC composites (0.30/0.32). The adapted health locus of control composites showed low internal consistency and were therefore interpreted with caution.

Post-stratification: We expanded the explanation of the weighting procedure. Post-stratification weights were calculated as the ratio between population proportions (derived from Austrian National Council election results) and observed sample proportions for each political category. The limitations of this procedure are acknowledged.

Pre-registration and power analysis: The manuscript now explicitly acknowledges that the study was not preregistered and no formal a priori power analysis was conducted. The analyses are accordingly interpreted as exploratory.

Measurement limitations: We added a note that several abbreviated or adapted multi-item measures showed only low to moderate internal consistency, which may have attenuated associations.

Comment 1.4 – Results

The results section is unduly descriptive. Tables and figures are not adequately interpreted. A stronger focus on effect sizes is needed.

We substantially revised the Results section. It is now organized along the research question clusters. Demographic composition has been moved to Methods. Single-item results have been removed. For all regression analyses, we now report regression coefficients (B), standard errors (SE), t-values, p-values, 95% confidence intervals, and model fit indices (R²). Four regression tables have been added to the manuscript. Effect sizes are reported where appropriate.

Comment 1.5 – Discussion

The discussion is excessively verbose and occasionally repetitious. There is a risk of overgeneralization beyond the correlational nature of the data.

The Discussion has been substantially tightened. All causal language (“why”) has been replaced with correlational framing (“what” and “how”). The key finding—the association between authoritarian orientation and perceived scientific validity of AM—now receives expanded treatment, including a new paragraph on the dissociation between epistemic judgment and behavioral choice. A reflexivity paragraph has been added. The term “interdisciplinary” has been replaced with “socio-psychological inquiry” where referring to this study’s methods. Three redundant closing paragraphs have been removed.

Comment 1.6 – References

Please modify and check reference styles per the journal guidelines.

Done. All references have been reformatted to PLOS ONE citation style.

Reviewer #2

We are grateful for Reviewer 2’s enthusiastic and incisive engagement with our work. Their comments—particularly regarding epistemological reflexivity and the authoritarianism finding—have meaningfully deepened the revised manuscript.

Comment 2.1 – Raw Data Availability

Please ensure all raw data is made publicly available.

See our response to Journal Requirement 2. The anonymized dataset can be obtained from the corresponding author upon reasonable request. We are clarifying institutional requirements for public repository deposit.

Comment 2.2 – General Assessment

Ultimately an incredibly fun manuscript to review. I am excited to see it get cleaned up to be even stronger.

We sincerely appreciate this generous assessment and have aimed to honor the reviewer’s comprehensive engagement by addressing each point carefully.

Comment 2.3 – AM Popularity, Benefits, and Conceptual Definitions

It is unclear why or how this form of medicine has become popularized. Concepts are introduced without definition or explanation.

The revised Introduction now explains AM’s popularity through institutional integration, holistic framing, and broader cultural visibility; defines Steiner’s Geisteswissenschaft as supersensible cognition cultivated through meditative practice; specifies the epistemological incompatibilities; and names the core metaphysical commitments (etheric bodies, formative forces, karmic processes). The climate change reference has been removed.

Comment 2.4 – Nazi/AM Connection

Clarify the epistemological parallelisms between AM and Nazism. Which antisemitic and racist beliefs were present in Steiner’s writings?

The passage now foregrounds structural-epistemological parallels: hierarchical cosmology, mystified epistemic authority, resistance to critical-rational inquiry. Steiner’s racist and antisemitic writings are cited with primary sources [24, 25]. The Heydrich quotation has been removed; the Adorno/Benjamin/Bloch framework has been retained and tightened.

Comment 2.5 – CAM Acronym

You define the CAM acronym in the abstract but not in the introduction.

Done. “Complementary and alternative medicine (CAM)” is now introduced at first use in the main text.

Comment 2.6 – Cronbach’s Alpha

Please perform and report Cronbach’s Alphas for each measure used.

Cronbach’s α has been calculated for all multi-item measures and is reported in the Methods section. See our response to Comment 1.3 for the values.

Comment 2.7 – Sociodemographic “Scores” and Statistical Tests

What are the “scores” you are referencing? Age is continuous while gender is categorical.

We revised the manuscript to clearly distinguish between continuous and categorical variables. The Methods section now specifies independent-samples t-tests for two-group comparisons of continuous variables, one-way ANOVA for more than two groups, Mann-Whitney U or Kruskal-Wallis tests for non-normally distributed variables, and chi-square or Fisher’s exact tests for categorical variables. Sociodemographic variables are treated according to their measurement level.

Comment 2.8 – P-Value Standardization

Please standardize your reporting of p-values to not go below p < .001.

Done. All p-values standardized throughout.

Comment 2.9 – Explicit Test Labels and Statistics

Please make clear which test was performed and ensure all necessary values are reported.

Each statistical test is now explicitly named. Regression results include B, SE, t-values, 95% CIs, p-values, and R². Demographic table footnotes specify whether chi-square or Fisher’s exact tests were applied.

Comment 2.10 – Demographics in Methods

Description of the demographic composition would fit more neatly within the method section.

Agreed. Demographic description has been moved to Methods.

Comment 2.11 – Forest Plots

The forest plots do not yet help illustrate your results. It is unclear which independent variables are included.

Forest plots have been revised with clearer labeling. The label in Figure 4 has been corrected from “Fascist tendencies” to “Authoritarian Attitudes (KSA-3).”

Comment 2.12 – Remove Single-Item Results

Please remove any results pertaining to specific items.

Agreed. All results based on individual survey items have been removed. Only scale-level findings are reported.

Comment 2.13 – Post-Stratification Justification

Please justify your inclusion of post-stratification techniques.

The Methods section now includes a dedicated justification. The distributional skew in political party affiliation indicated the sample was not representative. Population targets were derived from official Austrian National Council election results. Weights were calculated as the ratio between population and sample proportions per political category. The procedure and its limitations are now described in detail.

Comment 2.14 – Results Organization

Consider organizing based on the presented clusters of research questions.

Done. Results reorganized along the research question clusters.

Comment 2.15 – Duplicate Sentence

The first sentence in the discussion section repeats itself.

Done. Corrected.

Comment 2.16 – Causal Language

Be careful introducing questions of “why.”

All instances of “why” in the Discussion have been replaced with “what” and “how.”

Comment 2.17 – “Interdisciplinary”

I do not think “interdisciplinary” is an apt word to describe this study.

Agreed. References to this study as “interdisciplinary” have been replaced with “socio-psychological inquiry.”

Comment 2.18 – “Cross-Sectional” Framing

I do not find it appropriate to call this study “cross-sectional.”

We have removed “cross-sectional” as a design descriptor from the Discussion. Age is positioned as a covariate rather than a design element.

Comment 2.19 – “Could Be Determined as Significant”

The phrasing that these variables “could be” significant is vague.

Replaced with precise statistical reporting: “In the unweighted model, low ambiguity tolerance (B = 1.66, p = .013), prioritization of health optimization (B = 1.78, p < .001), and authoritarian attitudes (B = 1.72, p = .015) were each significantly associated with greater CAM endorsement.”

Comment 2.20 – CAM Preference and Lower Stress Tolerance

I would like to see a stronger interpretation of this association.

The revised Discussion considers several mechanisms: CAM’s longer consultations and participatory therapeutic relationship may offer greater patient agency; the holistic framing may reduce situational distress; conventional medicine’s probabilistic reasoning may be particularly aversive for individuals with low stress tolerance. These are framed as interpretive hypotheses requiring further investigation.

Comment 2.21 – Non-Significant Authoritarianism Result

Please note any potential statistical or methodological limitations. Maybe there are stronger ways to measure authoritarianism.

A dedicated paragraph addresses: (a) the KSA-3’s limitations as a brief screening instrument; (b) shared variance absorbed by post-stratification with political affiliation; (c) whether authoritarianism requires updated operationalization; and (d) the critical distinction that non-significance pertains to CAM preference, while the association with perceived scientific validity of AM remained robust (p < .001).

Comment 2.22 – Reflexivity Paragraph

I think it’s critically necessary to recognize that you are performing research using positivist methods that reproduce this very same episteme.

A reflexivity paragraph has been added drawing on the sociology of scientific knowledge (Bloor, 1991 [42]). We acknowledge that positivist-empirical methods operate within the same regime of epistemic authority whose dynamics we investigate. The symmetry principle is applied reflexively. We conclude that the study is situated as an intervention within, rather than an adjudication upon, the field of competing knowledge claims.

Comment 2.23 – Authoritarianism and Scientific Validity: Go Deeper

The key finding appears to be the association between authoritarianism and perceived scientific validity. This should fill up a larger portion of your discussion.

The revised Discussion gives this finding substantially expanded treatment: (a) the dissociation between robust association with perceived scientific validity (p < .001) and non-significant link with actual AM use (p = 0.536); (b) “scientific validity” as a marker of epistemic trust shaped by perceived source authority rather than evidence engagement; and (c) connections to the Adorno/Frenkel-Brunswik tradition and its contemporary relevance.

Attachment Submitted filename: Response_to_Reviewers_final.docx

# Peer review: Decision Letter 1

**DOI:** 10.1371/journal.pone.0348672.r003

**Authors:** Jiajia Ye

21 Apr 2026

<p>Authoritarian Attitudes and the Perceived Scientific Legitimacy of Anthroposophic Medicine: A Survey of Attitudes on Complementary and Alternative Medicine in Austria

PONE-D-25-42101R1

Dear Dr. Sitte,

We’re pleased to inform you that your manuscript has been judged scientifically suitable for publication and will be formally accepted for publication once it meets all outstanding technical requirements.

Within one week, you’ll receive an e-mail detailing the required amendments. When these have been addressed, you’ll receive a formal acceptance letter and your manuscript will be scheduled for publication.

An invoice will be generated when your article is formally accepted. Please note, if your institution has a publishing partnership with PLOS and your article meets the relevant criteria, all or part of your publication costs will be covered. Please make sure your user information is up-to-date by logging into Editorial Manager at Editorial Manager® and clicking the ‘Update My Information' link at the top of the page. For questions related to billing, please contact billing support.

If your institution or institutions have a press office, please notify them about your upcoming paper to help maximize its impact. If they’ll be preparing press materials, please inform our press team as soon as possible -- no later than 48 hours after receiving the formal acceptance. Your manuscript will remain under strict press embargo until 2 pm Eastern Time on the date of publication. For more information, please contact onepress@plos.org.

Kind regards,

Jiajia Ye

Guest Editor

PLOS One

Additional Editor Comments (optional):

Reviewers' comments:

Reviewer's Responses to Questions

**Comments to the Author**

1. If the authors have adequately addressed your comments raised in a previous round of review and you feel that this manuscript is now acceptable for publication, you may indicate that here to bypass the “Comments to the Author” section, enter your conflict of interest statement in the “Confidential to Editor” section, and submit your "Accept" recommendation.

Reviewer #1: All comments have been addressed

Reviewer #2: All comments have been addressed

**********

2. Is the manuscript technically sound, and do the data support the conclusions?

The manuscript must describe a technically sound piece of scientific research with data that supports the conclusions. Experiments must have been conducted rigorously, with appropriate controls, replication, and sample sizes. The conclusions must be drawn appropriately based on the data presented.

Reviewer #1: Yes

Reviewer #2: Yes

**********

3. Has the statistical analysis been performed appropriately and rigorously?

Reviewer #1: Yes

Reviewer #2: Yes

**********

4. Have the authors made all data underlying the findings in their manuscript fully available?

The PLOS Data policy requires authors to make all data underlying the findings described in their manuscript fully available without restriction, with rare exception (please refer to the Data Availability Statement in the manuscript PDF file). The data should be provided as part of the manuscript or its supporting information, or deposited to a public repository. For example, in addition to summary statistics, the data points behind means, medians and variance measures should be available. If there are restrictions on publicly sharing data—e.g. participant privacy or use of data from a third party—those must be specified.

Reviewer #1: Yes

Reviewer #2: Yes

**********

5. Is the manuscript presented in an intelligible fashion and written in standard English?

PLOS ONE does not copyedit accepted manuscripts, so the language in submitted articles must be clear, correct, and unambiguous. Any typographical or grammatical errors should be corrected at revision, so please note any specific errors here.

Reviewer #1: Yes

Reviewer #2: Yes

**********

6. Review Comments to the Author

Please use the space provided to explain your answers to the questions above. You may also include additional comments for the author, including concerns about dual publication, research ethics, or publication ethics. (Please upload your review as an attachment if it exceeds 20,000 characters)

Reviewer #1: I read the manuscript again. The authors' editing efforts are praiseworthy. I believe the authors have addressed every query raised in the initial review. The manuscript has been revised to improve its comprehensiveness and readability. It now has a better technical fit. The current state of the manuscript has the potential to advance knowledge, in my opinion.

Reviewer #2: Thank you for these thoughtful and thorough revisions. All concerns were addressed in this draft, making it suitable for publication.

**********

7. PLOS authors have the option to publish the peer review history of their article (what does this mean?). If published, this will include your full peer review and any attached files.

If you choose “no”, your identity will remain anonymous but your review may still be made public.

**Do you want your identity to be public for this peer review?** For information about this choice, including consent withdrawal, please see our Privacy Policy.

Reviewer #1: **Yes:** Gyanesh Kumar Tiwari

Reviewer #2: No

**********
